# Divergent recovery trajectories after mild traumatic brain injury are characterized by distinct acute profiles of neurofilament light, 4R-tau, and white matter diffusivity

**DOI:** 10.1007/s00415-026-14060-0

**Published:** 2026-08-25

**Authors:** Melissa G. Papini, Jacinta Thorne, Aleksandra K. Gozt, Andre N. C. Avila, Caerwen S. Ellery, Ruby Gilroy, Geena Gill, Francesca Buhagiar, Elizabeth Thomas, Amanda Armstrong, Steve Pedrini, Kevin Taddei, Ralph N. Martins, Alexander Ring, Glenn Arendts, Antonio Celenza, John Charles Iliff, Dan Xu, Stephen Honeybul, Gill Cowen, Ben Smedley, Daniel Fatovich, Michael Bynevelt, Carmela Pestell, Melinda Fitzgerald, Sarah C. Hellewell

**Affiliations:** 1 Curtin School of Diagnostic and Therapeutic Sciences, Curtin University, Perth, Australia; 2 Curtin Medical Research Institute, Faculty of Health Sciences, Curtin University, Perth, Australia; 3 Perron Institute of Neurological and Translational Science, Perth, Australia; 4 School of Allied Health, Faculty of Health Sciences, Curtin University, Bentley, WA Australia; 5 Connectivity Traumatic Brain Injury Australia, Nedlands, WA Australia; 6 Curtin Medical School, Faculty of Health Sciences, Curtin University, Bentley, WA Australia; 7 School of Psychological Science, The University of Western Australia, Perth, Australia; 8 School of Population Health, Curtin University, Bentley, WA Australia; 9 School of Medicine, The University of Western Australia, Nedlands, WA Australia; 10 Emergency Department, Fiona Stanley Hospital, Perth, Australia; 11 Division of Emergency Medicine, School of Medicine, The University of Western Australia, Perth, Australia; 12 Royal Flying Doctor Service-Western Operations, Jandakot, WA Australia; 13 Emergency Department, Saint John of God Hospital Murdoch, Perth, Australia; 14 Emergency Department, Royal Perth Hospital, Perth, Australia; 15 Emergency Department, Sir Charles Gairdner Hospital, Perth, Australia; 16 Emergency Department, Rockingham General Hospital, Perth, Australia; 17 The First Affiliated Hospital, Sun Yat-Sen University, Guangzhou, China; 18 Department of Neurosurgery, Sir Charles Gairdner Hospital, Perth, WA Australia; 19 Centre for Clinical Research in Emergency Medicine, Harry Perkins Institute of Medical Research, Perth, Australia; 20 Neurological Intervention and Imaging Service of Western Australia, Sir Charles Gairdner Hospital, Perth, Australia

**Keywords:** Mild traumatic brain injury, Post-concussion symptoms, Blood biomarkers, Diffusion magnetic resonance imaging, Psychological resilience, Coping skills

## Abstract

**Objectives:**

Blood-based and diffusion MRI (dMRI) biomarkers of white matter injury after acute mild traumatic brain injury (mTBI) were examined to: (1) distinguish mTBI from healthy controls (HC); (2) quantify inter-modality relationships; (3) identify associations between acute biomarker changes and recovery; (4) evaluate moderating influences of resilience and coping style.

**Methods:**

36 adults with mTBI (37.6±13.7 years; 37.1% female) and 35 HC participants (34.2 ± 11.9; 47.1% female) underwent baseline testing (≤ 9 days post-mTBI) including: blood-sampling (neurofilament light-chain [NfL], brain-derived tau, glial fibrillary acidic protein, ubiquitin carboxyl terminal hydroxylase-L1, four-repeat tau [4R-tau]), dMRI analysis of 48 white matter tracts, symptom (Post-Concussion Symptom Scale [PCSS]) and psychological questionnaires (Brief Resilience Scale, Utrecht Coping List). Recovery was determined at 3-, 6- and 12-month follow-ups using PCSS scores. Associations were evaluated using correlations, logistic regression, and exploratory moderation analyses. Trial registration Number: ACTRN12619001226190.

**Results:**

37.1% of participants reported persistent symptoms at 3 months, 42.9% and 28.6% at 6 and 12 months, respectively. NfL and 4R-tau were acutely elevated post-mTBI, alongside widespread increases in radial, mean, and axial diffusivity. Divergent recovery trajectories were defined by distinct baseline biomarker profiles: elevated NfL and mean diffusivity characterized recovered participants, while elevated 4R-tau and radial diffusivity characterized those who remained symptomatic. NfL was associated with recovery at 3 months and 4R-tau with symptomatic status at 12 months. Coping style, not resilience, moderated blood biomarker–outcome relationships.

**Conclusion:**

Divergent acute profiles of blood biomarkers, white matter diffusivity, and coping style distinguish mTBI recovery trajectories, providing hypothesis-generating support for biologically informed molecular, microstructural, and psychological mTBI phenotypes.

**Trial registration number:**

ACTRN12619001226190 approved March 2023.

**Supplementary Information:**

The online version contains supplementary material available at https://doi.org/10.1007/s00415-026-14060-0.

## Introduction

Approximately half the global population will experience one or more traumatic brain injuries (TBI) during their lifetime [1], of which approximately 90% are classified as mild (mTBI) [1]. mTBI is not readily captured using current management criteria due to diverse and dynamic symptom presentation, heterogeneity in the underlying pathophysiological processes engaged, and variable clinical outcomes [2–4]. Symptoms spanning physical, vestibular–ocular, cognitive, emotional/behavioral, and sleep domains [5–8] may be transient or evolve and develop over time, and are commonplace in the general population [9, 10]. Diagnostic and prognostic modeling are, therefore, complicated by concurrent injuries, pre-existing physical or mental health conditions, age-related comorbidities, or psychosocial contexts wherein family or domestic violence co-occur with the injury. This is particularly true in uncomplicated mTBI, defined by the absence of neurological trauma on standard neuroimaging including computed tomography (CT) and magnetic resonance imaging (MRI) scans (e.g., intracranial fracture, subdural hemorrhage), where diagnosis depends largely on symptom report and clinical judgment. Although many individuals spontaneously recover within a few weeks, Persisting Post-Concussion Symptoms (PPCS) describe symptoms that fail to resolve within three months of injury [11, 12]. Though PPCS prevalence estimates vary substantially (20–50%)^13^–^15^ owing to differences in how the term is operationalized, and inherent subjectivity of symptom self-reporting [16]. However, this uncertainty does not diminish its clinical significance. Given the high incidence of mTBI and the prolonged course of PPCS in some individuals, even conservative estimates translate to a substantial and burgeoning public health burden. The specific etiology of PPCS remains unclear. Acute structural and functional neuroimaging changes, elevations in blood-based biomarkers of neural injury, and pre-existing psychological health diagnoses have been linked to PPCS development [17–20]. Inconsistencies in subjective reports have shifted research focus toward incorporation of objective biomarkers to complement symptom reporting, elucidate pathology and inform recovery trajectories.

Although WM damage in uncomplicated mTBI is typically undetectable on routine clinical neuroimaging. Diffusion MRI (dMRI) uses the translational movement of water molecules as a proxy for WM architecture and is sensitive to post-injury microstructural alteration [21]. dMRI provides region-specific measures of molecular diffusion magnitude (diffusivity) and its directionality along an axis (anisotropy), offering insights into WM organization, axonal density, and osmotic change following injury [22–24]. In healthy individuals with coherent, myelinated WM tracts, fractional anisotropy (FA; diffusion directionality) tends to be higher, whereas mean diffusivity (MD; average magnitude of diffusivity in all directions) tends to be lower [22]. Axial diffusivity (AD), diffusion along the principal fiber direction, and radial diffusivity (RD; diffusion perpendicular to the principal fiber direction) are also reported, but less frequently investigated [22, 25, 26]. Together, both the magnitude and direction of these metrics change dynamically following injury, reflecting evolving microstructural and fluid-related processes. Previous work has identified long WM projection fibers as particularly vulnerable to mTBI, with acute increases in edema progressing to axonal loss in chronic stages, though findings remain variable [27–29]. Substantial heterogeneity in injury mechanism, pre-injury health, and inter-individual differences in the progression of WM changes over time likely obscure consistent group-level patterns and complicate the link between diffusion abnormalities and outcome trajectory. Furthermore, diffusion changes are sensitive, but not biologically specific, and in isolation provide limited insights into the molecular processes that underpin microstructural change.

Rapid acceleration–deceleration forces stretch and deform axons and cerebral blood vessels [30, 31], triggering a secondary cascade of biochemical responses including cytoskeletal breakdown, axolemmal disruption, and impaired axonal transport [32–35]. Axons are particularly susceptible to secondary injury mechanisms that can propagate damage across their length and disrupt white matter (WM) tracts distal to the injury site [34–36]. This progressive secondary WM alteration is thought to significantly contribute to the development and persistence of symptoms [35, 37, 38]. Given the diverse and evolving nature of secondary injury processes, robust blood-based and neuroimaging biomarkers that are proportionate to the injury, biologically relevant, and feasible in clinical settings are essential to characterize mTBI, guide the development of diagnostic and prognostic clinical tools and provide objective, quantifiable indices that complement symptom-based assessment. However, biomarker findings derived from any single domain often show inconsistency across studies; addressing these limitations necessitates a complementary multimodal approach. Incorporating neuroimaging alongside blood-based biomarkers allows specific regions of pathology to be identified in parallel with changes in multiple blood biomarkers to better characterize heterogeneous injury mechanisms and strengthen links between underlying pathophysiology and symptom presentation [27]. The strength and direction of biomarker-outcome relationships may be influenced by individual-level biopsychosocial factors termed modifiers [2, 39]. Interpreting these relationships requires a highly nuanced understanding of the temporal pathophysiology that accompanies mTBI. Consistent with this, updated diagnostic criteria by the American Congress of Rehabilitation Medicine (ACRM) recommend inclusion of neuroimaging and blood-based biomarker assessment for mTBI, alongside consideration of contextual factors [40]. The Clinical assessment, Blood-based biomarkers, Imaging, and Modifiers (CBI-M) framework formalizes this approach by integrating biological measures with patient specific biological, psychological, sociological, and ecological factors [2, 3, 39, 41]. Modifiers may influence recovery by shaping behavioral and physiological responses to injury, as well as the way symptoms are perceived and managed [42, 43]. Psychological resilience, the capacity to cope with and positively adapt to stressful situations, has emerged as a clinically relevant and potentially malleable factor associated with TBI recovery [41, 44–46]. Resilience and coping style are closely related constructs arising from a constellation of dispositional traits, such as optimism and self-efficacy, and situational factors, including support systems and prior life experiences [47, 48]. Leventhal’s Common-Sense Model (CSM) of Self-Regulation [49] posits that individuals construct mental representations of illness across five dimensions: identity, cause, timeline, consequences, and control. Following mTBI, a resilient individual may view their symptoms (identity) as temporary (timeline), manageable (control), and an expected consequence of injury (cause), with minimal disruption to daily life (consequences). This interpretation is likely to support adaptive coping [50–52]. In contrast, individuals with lower resilience may perceive the same symptoms as severe, prolonged, and uncontrollable, reinforcing maladaptive coping and increasing the risk of PPCS [43, 53, 54]. The extent to which these internal attributes exert a quantifiable influence on biomarkers and alter the trajectory of recovery trajectories is unknown. Previous reports linking resilience with preservation of WM microstructure following mTBI suggest an under-recognized biological effect [55, 56] though no relationship to blood-based biomarkers has been documented. Establishing such relationships may strengthen inference regarding pathways through which modifiers influence outcome and support the development of proactive, pre-emptive treatment strategies that address recovery from both psychological and biological perspectives.

This study employs both blood and diffusion imaging biomarkers within a clinically representative mTBI cohort to address and clarify relationships between acute WM changes defined by dMRI and blood biomarker changes, with resilience, coping style and the development of persisting symptoms. Specifically, this study aimed to: (1) Determine whether acute blood and dMRI biomarkers of WM injury differ between individuals with mTBI and age- and sex-matched healthy controls (HC); (2) Quantify the relationship between WM blood and diffusion biomarkers relative to recovery; (3) Investigate whether acute biomarkers are associated with longitudinal recovery trajectory; (4) Conduct exploratory analyses to determine whether resilience or coping strategies in the acute-phase influence symptom recovery and WM pathology following mTBI.

## Methods

### Study design

This study used data collected from a subset of participants enrolled in the Concussion REcovery STudy (CREST)^8^, a prospective, longitudinal observational study investigating predictors of mTBI recovery conducted in Perth, Western Australia. Ethics approval was obtained from Royal Perth Hospital Human Research Ethics Committee (#RGS0000003024), Curtin University (HRE2019-0209), Ramsay Health Care (#2009), St John of God Health Care (#1628) and registered on the Australian New Zealand Clinical Trials Registry (ACTRN12619001226190). All participants provided informed written consent prior to participation.

Recruitment occurred via referrals from hospital emergency departments, general practitioners (GPs), allied health professionals, sports physicians, or self-referral. The mechanism of injury was intentionally broad to capture the full spectrum of mTBI. Eligible participants were aged 18–65 years and in the previous seven days, diagnosed by a qualified medical practitioner. Participants had uncomplicated mTBI, defined as the absence of acute trauma-related intracranial abnormality visible on CT or conventional MRI, inclusive of, but not limited to: intracranial hemorrhage, cerebral contusion or depressed skull fracture. Further, inclusion required a period of post-traumatic amnesia (PTA) <24 h, loss of consciousness (LOC) <30 min and symptoms attributable to the injury at recruitment. Participants were additionally excluded if they had a Glasgow Coma Score <14 at initial assessment; a significant history of pre-existing conditions that would interfere with outcome assessment or follow-up (e.g., substance or alcohol abuse, homelessness, terminal illness); a significant debilitating pre-existing mental health disorder (e.g., schizophrenia, bipolar disorder); a significant pre-existing neurological condition (e.g., stroke, dementia); pre-existing cognitive impairment, such as an intellectual disability; non-English speaking or poor English-language skills; pregnancy; or health issues that contraindicated the safety of MRI protocols.

CREST comprises two phases. All enrolled participants completed Phase I: a semi-structured telephone interview within seven days of injury to assess eligibility and collect demographic, injury and acute case data. Participants able to attend in-person were eligible for baseline testing, conducted at Curtin University and the Perron Institute for Neurological and Translational Science facilities (Ralph and Patricia Sarich Neuroscience Research Institute, Nedlands, Western Australia). Baseline testing comprised blood-sampling, neuropsychological testing and symptom questionnaires, completed within seven days of injury, and MRI, acquired within nine days of injury; the longer time window due to limited availability of the clinical MRI scanner [57]. Participants were followed up via telephone at one, three, six- and 12-months post-injury to determine symptomatic recovery. Age- and sex-matched healthy controls (HC) without mTBI in the previous five years were also recruited for Phase II only. A detailed description of the CREST testing procedure, inclusion and exclusion criteria is described in detail by Gotz et al. [57].

### Questionnaires

Participants completed questionnaires in a private room under researcher supervision and took approximately 30–40 min to complete. Post-concussion symptom number and severity were assessed using the Post-Concussion Symptom Scale (PCSS), a 22-item self-report measure rated on a 7-point Likert scale (0 = none to 6 = severe, total range = 0–132). Symptom number was calculated as the count of items scored >0. At each follow-up, participants were classified as recovered if total PCSS scores were <6 for males and <7 for females [5, 58] and as symptomatic otherwise. Psychological resilience was assessed using The Brief Resilience Scale (BRS), a validated 6-item questionnaire using a 5-point Likert scale (1=strongly disagree to 5=strongly agree; mean score: 0–5; higher scores indicate greater resilience; alpha = 0.80–0.91) [59]. Coping style was assessed using the English version of the Utrecht Coping List (UCL) [60–62], a 47-item self-report measure evaluating coping behavior frequency over the preceding three months (1 = seldom/never to 4 = very often). Forty-four items contribute to seven subscales: active confronting, palliative reaction, avoidance, seeking social support, passive reaction pattern, expressing emotions, and reassuring/comforting thoughts. Subscale scores were standardized to a 1–4 scale by dividing the sum by the number of items [63]; higher scores indicate a greater preference for each specific coping style. Both the BRS and UCL were only collected at baseline; the PCSS was collected at baseline and repeated at each follow-up.

### Blood-based biomarkers

Non-fasting venous blood samples were collected at baseline by a researcher trained in phlebotomy. Approximately 20 mL of whole blood was drawn into BD Vacutainer™ EDTA plasma and SST™ serum separation tubes (BD, Franklin Lakes, NJ), centrifuged at 3000 rpm at 4 °C for 10 min; aliquots (250 µL) were stored at − 80 °C until analysis.

Serum 4R-tau was quantified using the Abcam SimpleStep® colorimetric sandwich ELISA® kit (Abcam, Cambridge, UK; ab309282) per manufacturer’s instructions. Values below the limit of detection (LOD: 5.26 pg/mL) were treated as zero; mean intra-assay coefficient (CV) of variation was 11.3%. Plasma concentrations of NfL, BD-Tau, UCH-L1, and GFAP were measured using the Simoa® Neurology 4-Plex D Advantage Plus kit (Quanterix, Lexington, MA;104,822) on the HD-X Analyser™, with samples diluted fourfold. All assays were run in duplicate with two manufacturer-provided controls per plate. The LODs for NfL, BD-tau, UCH-L1 and GFAP were 0.094 pg/mL, 0.029 pg/mL, 0.577 pg/mL and 0.121 pg/mL, respectively. Average intra-assay CV for NfL, BD-tau and GFAP were 9%, 4.9% and 3.6%, respectively. UCH-L1 was excluded from analyses due to high intra-assay variability and a substantial proportion of imprecise or missing values.

### MRI analysis

MRI scans were acquired within 9 days of injury at the Department of Radiology at Sir Charles Gardiner Hospital (Nedlands, WA), using a Philips Ingenia 3 T Multi-Transmit Wide Bore Scanner (Phillips Healthcare, Best, Netherlands) with a 32-channel head coil. High-resolution T1-weighted structural images were acquired with an echo time/repetition time (TE/TR) of 2.7/6.01 ms, a Field of View (FOV) of 256 mm, and a matrix size of 256 × 256. A total of 175 slices were obtained at 1 mm isotropic resolution, with a scan duration of 6 min and 19 s. dMRI data were acquired using a single-shell protocol with 32 diffusion-encoding directions at a *b*-value of 1000 s/mm2, along with a single non-diffusion-weighted (b = 0 s/mm2) volume with a TE of 113 ms and TR of 4694 ms. The acquisition was performed with a 224 mm FOV, a matrix size of 110 × 110, and whole-brain coverage comprising 60 slices with 2 mm isotropic resolution. The total acquisition time for the diffusion-weighted sequence was 11 min and 36 s. Full imaging protocol, described previously [57], took approximately 50-min and all scans were reviewed for abnormalities by a neuroradiologist (MB), with incidental findings referred to participants’ GP.

Image processing and analysis were conducted using Neurodesk (v2025-02–04) [64], a Linux-based virtual platform employing Docker containerisation (v4.38.4). Custom-built BASH processing scripts were used to automate analysis workflows, ensuring reproducibility and consistency across computational environments. Image conversion and pre-processing followed best-practice methods and all data were organized in accordance with the Brain Imaging Data Structure (BIDS) [65] standard. DICOM images were first converted into the Neuroimaging Informatics Technology Initiative (NIfTI). nii format using the dcm2nii tool [66, 67]. A visual inspection of all images was conducted to ensure images were free from artifacts and of sufficient quality for analysis. Pre-processing of raw diffusion-weighted imaging (DWI) data was conducted using the Functional Magnetic Resonance Imaging of the Brain (FMRIB) Software Library (FSL, v6.0.7.8) [68–70] and MRtrix3 (v3.3.0.4) [71, 72]. First, NIfTI images were converted to MRtrix-specific image format (.mif). Data were denoised, Gibbs ringing artifacts removed, corrected for eddy current and motion distortion and bias-corrected. Non-brain tissue was removed to generate whole-brain masks. Tissue-specific response functions for WM, gray matter (GM) and cerebrospinal fluid (CSF) were estimated with selected voxels and saved for quality assessment. Single-Shell 3-Tissue constrained spherical deconvolution was performed using MRtrix3Tissue (v5.2.8; https://3tissue.github.io/), a fork of MRtrix3 [71, 72], to estimate the white matter fiber orientation distribution function prior to spatial normalization. FSL’s DTIFIT tool (fsl.fmrib.ox.ac.uk/fsl/docs/#/diffusion/dtifit) [68–70] was used to estimate the diffusion tensor at each voxel.

Individual, pre-processed, eddy current corrected images along with corresponding b-values, b-vectors and brain masks were used to calculate FA, MD, AD and RD maps at each voxel. Region-of-interest (ROI) analysis was conducted using the Johns Hopkins University ICBM-DTI-81 White Matter atlas [73, 74] included in FSL. Each of the 48 white matter tracts (defined in Online Resource 1) were extracted as ROIs and converted into individual masks and values extracted for FA, MD, AD and RD enabling tract-specific quantification of the white matter microstructure of individual participants for further statistical analysis. For ease of visualization, MD, AD, and RD values were scaled by a factor of 10,000 (reported as ×10⁻4 mm2/s); FA values are unitless.

### Statistical analysis

Analyses were conducted in IBM SPSS (v.30, IBM Corporation, Armonk, New York, New York) and GraphPad Prism (V.10.4, GraphPad Software, Boston, Massachusetts). Normality of continuous variables was assessed via visual inspection of histograms and the Shapiro–Wilk test (where *p*>0.05).

Demographic, clinical, questionnaire, blood biomarker, and diffusion metric data were first compared between mTBI and HC groups and secondly between HC, recovered and symptomatic mTBI groups at each follow-up timepoint (3, 6, and 12 months). Continuous variables were analyzed using independent samples *t*-tests or Mann–Whitney U tests; categorical variables were compared using chi-squared tests. Three-group comparisons (HC, recovered mTBI, symptomatic mTBI) used one-way ANOVA or Kruskal–Wallis tests as appropriate, with pairwise post hoc comparisons corrected for multiple comparisons using the two-stage linear set-up false discovery rate (FDR) correction by Benjamin, Krieger and Yekutieli (Q=0.05). To account for small and uneven group sizes, bootstrapping (10,000 resamples) was applied to obtain robust *p*-value estimates and 99% confidence intervals. Outliers were identified prior to analyses using robust regression and outlier removal (ROUT) method (Q=1%).

Associations between blood biomarker concentrations and diffusion metrics were evaluated using two-tailed Pearson’s or Spearman’s rank-order correlations for normally distributed variables and for non-normally distributed variables, respectively. Analyses were restricted to measures showing significant group-level differences in preceding comparisons corrected for multiple comparisons using the Benjamini–Hochberg false FDR procedure. Correlations were repeated for recovered and symptomatic mTBI participants separately at each follow-up timepoint.

Candidate blood biomarkers and diffusion ROIs that significantly differed between mTBI and HC were entered into univariate binary logistic regression models to determine case–control discriminative capacity. Baseline biomarkers meeting this criterion that additionally differed significantly between recovered and symptomatic mTBI groups at a given follow-up timepoint were entered into additional logistic regression models to determine their association with symptomatic mTBI status at follow-up. Due to limited sample sizes and the need to preserve model stability, covariates were excluded with models using 10, 000 bootstrap resamples and being reported as bias-corrected and accelerated (BCa) 95% confidence intervals.

For candidate biomarkers that discriminated between symptomatic and recovered mTBI participants, exploratory moderation analyses were conducted (Hayes PROCESS macro, Model 1) [75] to evaluate whether psychological resilience or coping style influenced the relationship between biomarkers and longitudinal outcome in the mTBI group. Where significant interaction effects were probed using simple slopes analysis at ±1 standard deviation from the mean. Given the exploratory nature of these analyses, both significant and trend-level interactions are reported for hypothesis-generating purposes.

For all analyses, significance was set at *p* < 0.05. Missing data were handled on a case-by-case basis. Participants were included in each analysis if the relevant data were available, with listwise deletion applied to ensure consistency within each model and maximize the use of available data.

## Results: comparison of baseline mTBI vs. healthy controls

### Demographic and clinical characteristics

**Fig. 1:**
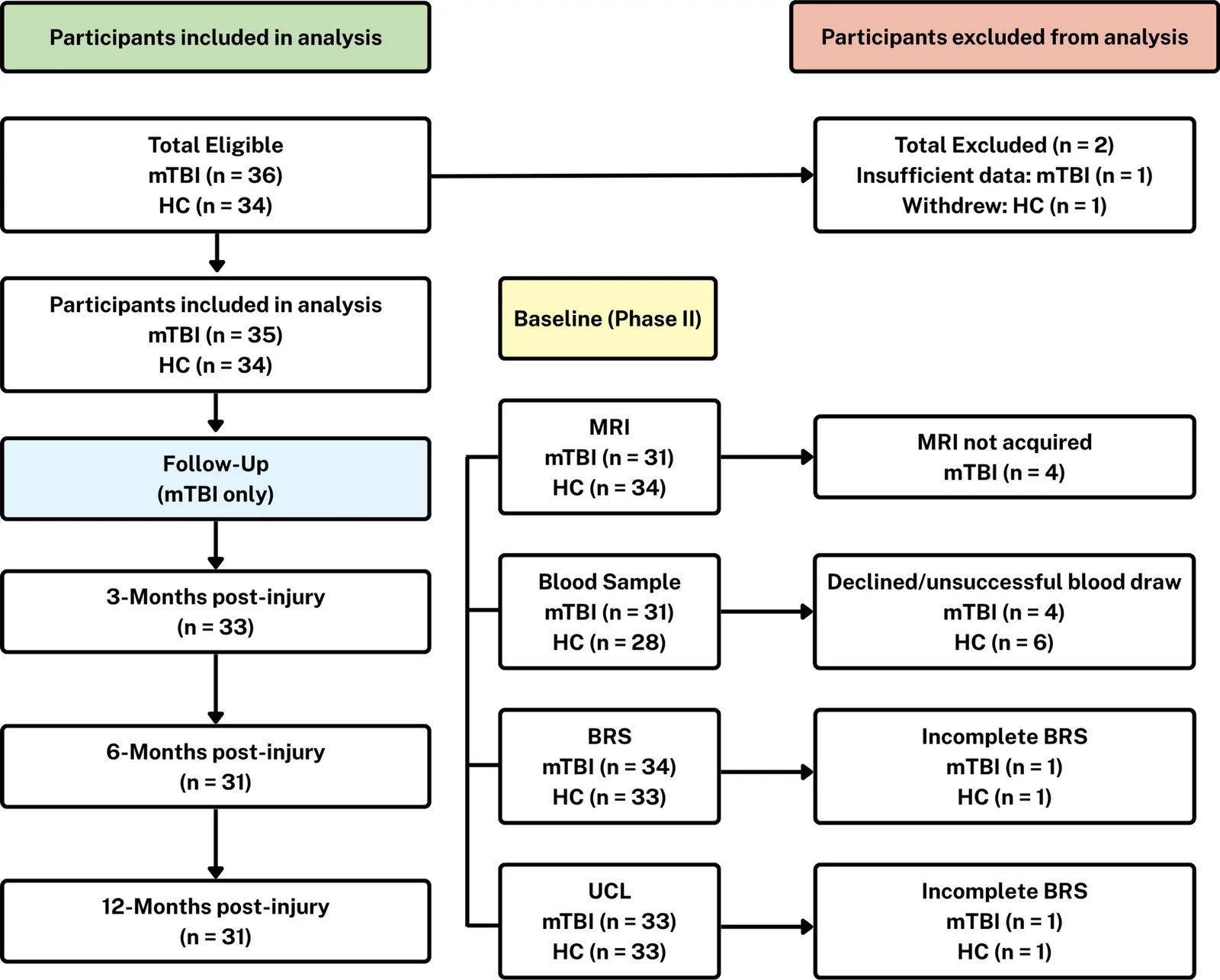
Flowchart of participants enrolled in CREST baseline. BRS, Brief Resilience Scale; HC, healthy control; mTBI, mild traumatic brain injury; UCL, Utrecht Coping List

Demographic details and injury characteristics are listed in Table 1. Of the 231 Phase I participants, 35 mTBI participants met eligibility criteria and were included for analysis; 34 age- and sex-matched healthy controls, without a history of mTBI in the previous five years (HC) were subsequently recruited (Fig. 1). For mTBI participants, blood samples (n = 31) were collected 5.5 ± 1.2 days post-injury and MRI scans (*n* = 31) performed at 6.8 ± 1.9 days. Follow-up telephone calls were conducted at 3 months (92.2 ± 4.7 days, *n *= 33), 6 months (185.7 ± 9.7 days, *n* = 31) and 12 months (372.9 ± 15.8 days, *n* = 31) post-injury. Age- and sex-matched HC data were collected from a single in-person visit. Our final total sample was aged 35.9 ± 12.9 years (range: 20.3–65.8 years) and comprised 42% female participants. 33 mTBI participants were recruited via ED and two were self-referred following diagnosis from a GP following a sporting injury and striking their head on an object. Additional demographic information, acute UCL and BRS findings are listed in Table 1; demographic information, follow-up PCSS scores, BRS and UCL scores split into follow-up recovery groups are listed in Online Resource 2.

**Table 1:** Acute mTBI and Healthy Control Demographic and Injury Characteristics

Mean ± SD or n (%)	mTBI (*n* = 35)	Healthy Controls (*n* = 34)	*p*-value
Demographics			
Sex (% female)	13 (37.1)	16 (47.1)	0.40
Age (years)	37.6 ± 13.7	34.2 ± 11.9	0.33
BMI	24.8 ± 2.8	23.1 ± 3.2	0.02*
Years of Education	14.0 ± 2.3	15.5 ± 1.8	0.03*
Days post-injury—Baseline	5.6 ± 1.1	-	-
Days post-injury—MRI	6.8 ± 1.8	-	-
Pre-existing conditions^†^			
Prior mTBI			
0	12 (34.3)	28 (82.4)	<0.001*
1–2	13 (37.1)	6 (17.6)	<0.001*
3+	10 (28.6)	0 (0)	<0.001*
Migraine	6 (17.1)	2 (5.9)	0.14
Dyslexia	2 (5.7)	0 (0)	0.16
ADHD	2 (5.7)	5 (14.7)	0.22
Insomnia	1 (2.9)	2 (5.9)	0.53
Sleep apnea	1 (2.9)	0 (0)	0.54
Depression	6 (17.1)	8 (23.5)	0.51
Anxiety	4 (11.4)	7 (20.6)	0.30
PTSD	1 (2.9)	0 (0)	0.32
Injury Characteristics			
Mechanism of injury			
Sports-related	20 (57.1)	-	-
Falls	8 (22.9)	-	-
MVA	6 (17.1)	-	-
Struck object	1 (2.9)	-	-
Loss of consciousness			
Yes	11 (31.4)	-	-
Unsure	9 (25.7)	-	-
Posttraumatic amnesia			
Yes	19 (54.3)	-	-
Unsure	1 (2.9)	-	-
Additional extracranial injury	17 (48.6)	-	-
Questionnaires			
Post-Concussion Symptom Scale			
Symptom severity	23.5 ± 20.2	7.1 ± 9.1	<0.001*
Symptom number	11.1 ± 5.9	3.9 ± 3.7	<0.001*
Brief Resilience Scale			
Average Score	3.8 ± 0.6	3.7 ± 0.6	0.32
Utrecht Coping List			
Active tackling	2.7 ± 0.4	3.0 ± 0.5	0.04*
Palliative response	2.3 ± 0.4	2.5 ± 0.6	0.09
Avoidance/waiting	2.1 ± 0.4	2.1 ± 0.4	0.81
Seeking social support	2.4 ± 0.6	2.5 ± 0.7	0.76
Passive response pattern	1.6 ± 0.5	1.7 ± 0.5	0.92
Expression of emotions	2.1 ± 0.5	2.1 ± 0.7	0.49
Reassuring thoughts	2.8 ± 0.5	2.7 ± 0.5	0.52

*ADHD* Attention-Deficit Hyperactivity Disorder, *BMI* Body Mass Index, *MVA* motor vehicle accident, *PTSD* Post-Traumatic Stress Disorder

Continuous variables are reported as mean ± SD; categorical variables as n (%). Normality was assessed using the Shapiro–Wilk test; p>0.05 was considered evidence of deviation from normality

^*^Bolded text depicts significant group differences (*p* < 0.05) evaluated using independent samples t-tests for normally distributed continuous variables and Mann–Whitney U tests for non-normally distributed continuous variables. Categorical variables were compared using chi-squared tests. ^***†***^Pre-existing variables including concussion history and medical history were self-reported

### Acute post-concussion symptoms

Baseline symptom burden was significantly greater in mTBI participants compared to HC for both symptom severity (PCSS total score: mTBI: 23.5 ± 20.2; HC: 7.1 ± 9.1; *p*<0.001) and symptom number (mTBI: 11.1 ± 5.9; HC: 3.9 ± 3.7, *p*<0.001).

### Resilience and coping style

Both BRS and UCL were administered at baseline (in-person testing ≤7d) only. Results of these questionnaires are outlined in Table 1; comparisons between baseline BRS and UCL scores by recovered and symptomatic mTBI groups at follow-up are listed in Online Resource 2. No significant differences were found in BRS scores between HC and mTBI groups (*p* > 0.05). For UCL subscales, mTBI participants reported a significantly reduced preference for active tackling (t(64) = −2.08, *p* = 0.04; mTBI: 2.7 ± 0.4; HC: 3.0 ± 0.5) with no significant differences between HC and mTBI groups for any other coping style (passive reaction response, avoidance, seeking social support, palliative response, expression of emotions, and reassuring thoughts).

### Blood biomarkers

Blood biomarker concentrations sampled at baseline (mean 5.6 days post-injury, Table 1) were compared between groups. Concentration of BD tau, NfL, GFAP and 4R-tau biomarkers are listed in Table 2 and visualized in Online Resource 4.

**Table 2:** Baseline blood concentrations of four-repeat tau, brain-derived tau, neurofilament light chain and glial fibrillary acidic protein stratified by group

Group	NfL	4R-tau		BD-tau	GFAP
Baseline					
Control	3.2 ± 1.8 (26)^a,b^	15.2 ± 11.2 (28)^a,b^		6.4 ± 1.3 (27)	54.2 ± 18.1 (27)
mTBI	10.1 ± 13.0 (31)^a^	24.3 ± 17.3 (30)^a^		6.1 ± 1.7 (31)	55.2 ± 23.3 (31)
3 Months					
Recovered	12.7 ± 15.0 (18)^b^	22.6 ± 13.8 (18)		6.1 ± 1.7 (18)	57.4 ± 26.0 (18)
Symptomatic	7.5 ± 10.0 (11)^c^	28.5 ± 22.9 (11)		6.3 ± 1.8 (11)	53.0 ± 18.5 (11)
6 Months					
Recovered	11.1 ± 14.2 (16)	21.3 ± 16.7 (16)	6.1 ± 1.8 (16)		53.8 ± 18.2 (16)
Symptomatic	10.4 ± 13.3 (12)	32.6 ± 17.2 (12)^b^	6.1 ± 1.7 (12)		52.4 ± 14.3 (12)
12 Months					
Recovered	11.4 ± 14.5 (20)^b^	19.0 ± 15.7 (20)^c^	6.1 ± 1.7 (20)		58.2 ± 25.7 (20)
Symptomatic	7.1 ± 9.8 (8)	38.3 ± 16.9 (8)^b,c^	6.0 ± 1.8 (8)		47.9 ± 15.4 (8)

*4R-tau* four-repeat tau isoform, *BD-tau* brain-derived tau, *GFAP* glial fibrillary acidic protein, *mTBI* mild traumatic brain injury, *NfL* neurofilament light chain, *PPCS* persisting-post-concussion symptoms

Data are presented in pg/mL as mean ± SD (n). Normality was assessed according to the Shapiro–Wilk test: *p* > 0.05. Baseline comparisons between mTBI and HC were performed using Mann–Whitney U tests. For 3-, 6- and 12-month trajectory groupings, differences between control, recovered mTBI and PPCS groups using Kruskal–Wallis tests and, where significant by Dunn post hoc pairwise comparisons with two stage Benjamini, Krieger, and Yekutieli false discovery rate correction (Q = 0.05). Statistical significance was set at *p* < 0.05

^a^Denotes differences between healthy control and mTBI;

^b^Denotes differences between healthy control and symptomatic mTBI or recovered mTBI groups;

^c^Denotes differences between recovered mTBI and symptomatic mTBI groups

### Acute post-injury NfL and 4R-tau elevation discriminate mTBI from HC

Compared to HC, mTBI participants had higher baseline NfL (U = 235, *p* = 0.01; Table 2). Baseline NfL concentrations significantly discriminated mTBI participants from controls: [χ2(1)=7.55, p=0.006, Nagelkerke R2=0.168] correctly classifying 62.5% of cases [Bootstrapped: B = 1.87, OR = 6.51, *p* = 0.008, BCa 95% CI (0.61, 4.26)]. In the mTBI group, 4R-tau concentrations were significantly higher than HC (U = 293.5, *p* = 0.049; Table 2) and were able to distinguish between groups in regression models. The overall model was significant [χ2(1)=5.56, *p* = 0.018, Nagelkerke R2 = 0.122] and correctly classified 60.3% of cases. Higher 4R-tau was associated with a marginally increased likelihood of being in the mTBI group [Bootstrapped: B = 0.045, OR = 1.05, *p* = 0.011, BCa 95% CI (0.009, 0.095)]. No differences were observed between mTBI and HC groups for BD-tau (*p* = 0.23) or GFAP (*p* = 0.92), with these markers not considered for further analysis.

### Diffusion MRI differences between HC and mTBI groups

**Fig. 2:**
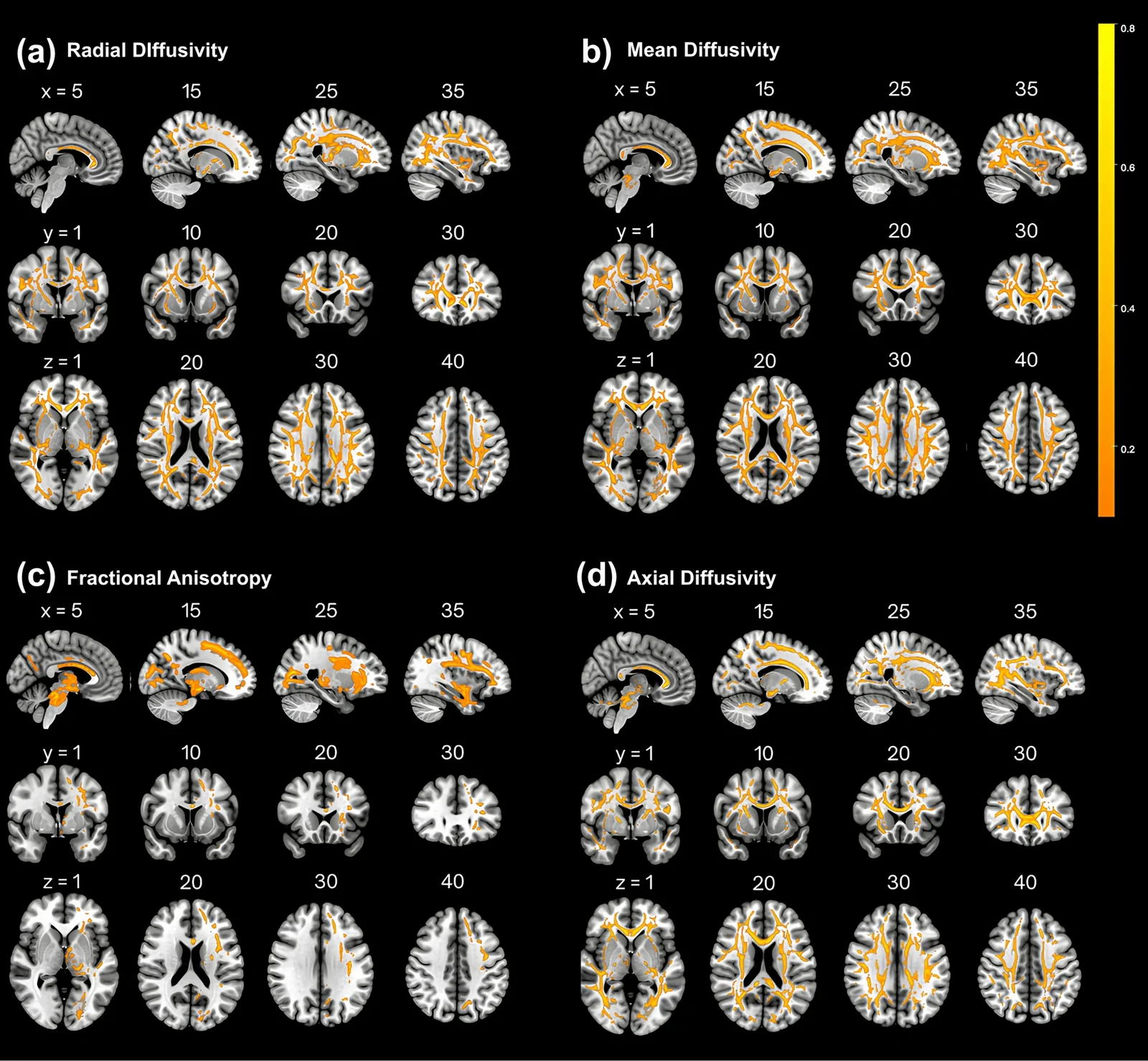
Tract-Based Spatial Statistics (TBSS) results showing regions of significant white matter microstructural differences between the mTBI and healthy control (HC) groups. t-statistic maps are displayed for **(a)** radial diffusivity (RD), **(b)** mean diffusivity (MD), **(c)** fractional anisotropy (FA), and **(d)** axial diffusivity (AD), overlaid on the MNI152 standard-space template in axial (top), coronal (middle), and sagittal (bottom) views. Significant voxels were thickened using the tbss_fill command for visualization purposes. The color bar indicates t-statistic values ranging from 0.2 to 0.8. Widespread differences were observed in RD, MD, and AD across major white matter tracts, while FA differences were more spatially restricted

Baseline diffusion metrics for each white matter tract were compared between mTBI and HC groups; significant differences are listed in Table 3. Broadly, mTBI participants exhibited widespread elevations in RD and AD across projection and association fibers alongside more limited decreases in FA and increases in MD compared to HC (Fig. 2).

**Table 3:** Diffusion MRI metrics showing significant group differences between mTBI and healthy controls across white matter tracts

Metric	Tract (Hemisphere)		HC (*n* = 34)	mTBI (*n* = 31)	Direction	*p*-value
Radial Diffusivity	PCR (L)	4.8 ± 0.45		5.07 ± 0.55	↑	0.023
	ACR (R)	4.74 ± 0.4		5.06 ± 0.61	↑	0.015
	RLIC (L)	4.04 ± 0.32		4.28 ± 0.43	↑	0.011
	SLF (L)	4.16 ± 0.27		4.28 ± 0.24	↑	0.007
	SLF (R)	4.17 ± 0.29		4.31 ± 0.25	↑	0.008
Mean Diffusivity	ACR (L)	6.47 ± 0.39		6.72 ± 0.51	↑	0.030
	ACR (R)	6.70 ± 0.39		6.95 ± 0.50	↑	0.022
	CGC (R)	6.70 ± 0.21		6.86 ± 0.33	↑	0.006
Fractional Anisotropy	SCR (L)	0.47 ± 0.03		0.46 ± 0.02	↓	0.044
	PCR (L)	0.45 ± 0.03		0.44 ± 0.03	↓	0.037
	EC (R)	0.42 ± 0.02		0.41 ± 0.02	↓	0.030
Axial Diffusivity	ACR (L)	9.99 ± 0.64		9.58 ± 0.43	↓	0.004
	ACR (R)	10.4 ± 0.84		9.90 ± 0.39	↓	0.002
	CST (R)	6.74 ± 0.46		6.46 ± 0.24	↓	0.011
	PTR (R)	12.9 ± 0.71		12.47 ± 0.55	↓	0.009
	SFO (L)	9.71 ± 0.44		9.45 ± 0.28	↓	0.017

*HC* healthy controls, *mTBI* mild traumatic brain injury, *PCR* posterior corona radiata, *ACR* anterior corona radiata, *RLIC* retrolenticular part of the internal capsule, *SLF* superior longitudinal fasciculus, *CGC* cingulum (cingulate gyrus), *SCR* superior corona radiata, *EC* external capsule, *CST* corticospinal tract, *PTR* posterior thalamic radiation, *SFO* superior fronto-occipital fasciculus, *L* left hemisphere, *R* right hemisphere

Mean ± SD values are presented for each group. Group comparisons were conducted using independent samples t-tests for normally distributed data and Mann–Whitney U tests for non-normally distributed data, with normality assessed using the Shapiro–Wilk test. Only tracts with significant between-group differences are shown. ↑ higher in mTBI; ↓ lower in mTBI. Units: AD, MD and RD in ×10⁻^4^ mm^2^·s⁻^1^; FA is unitless

### Diffusion ROIs discriminate mTBI from HC

Of the 48 tracts assessed, 12 showed significant between-group differences in RD, MD, FA and/or AD and were carried forward into univariate binary logistic regression to determine whether baseline diffusion metric discriminated mTBI from HC. Eleven of the 12 tracts yielded significant models (Table 4). The strongest models by variance explained were AD in the left ACR (Nagelkerke R2 = 0.177) and MD in the left SLF (R2 = 0.151). Lower FA in the right EC (R2 = 0.106) and left PCR (R2 = 0.095) significantly discriminated mTBI from HC.

**Table 4:** Univariate binary logistic regression for diffusion MRI metrics to discriminate acute mTBI from healthy controls

Metric	Tract	χ^2^(df)	P (Omnibus)	R^2^	Correct (%)	B (BCa)	*p* (BCa)	BCa 95% CI [lower, upper]
RD	ACR (R)	3.82 (1)	0.051	0.080	62.9	1.20	0.091	[− 0.21, 3.63]
	PCR (L)	4.65 (1)	0.031	0.095	63.5	1.14	0.038	[− 0.003, 2.91]
	RLIC (L)	5.14 (1)	0.023	0.105	58.7	1.80	0.024	[0.1, 4.53]
	SLF (R)	4.20 (1)	0.040	0.088	58.1	1.95	0.045	[0.07, 4.84]
	SLF (L)	3.38 (1)	0.066	0.072	57.4	1.88	0.083	[− 0.42, 5.65]
MD	ACR (L)	4.70 (1)	0.030	0.099	57.4	1.31	0.028	[− 0.07, 3.51]
	ACR (R)	4.77 (1)	0.029	0.103	63.3	1.35	0.039	[0.02, 3.92]
	CGC (R)	5.31 (1)	0.021	0.112	62.3	2.47	0.049	[− 0.05, 11.18]
	SLF (L)	7.28 (1)	0.007	0.151	59.0	2.47	0.009	[0.87, 5.89]
FA	SCR (L)	4.16 (1)	0.042	0.083	63.1	−21.7	0.061	[− 48.4, − 2.18]
	PCR (L)	4.82 (1)	0.028	0.095	66.2	−21.8	0.037	[− 46.6, − 4.20]
	EC (R)	5.30 (1)	0.021	0.106	62.5	−32.3	0.017	[− 59.9, − 9.45]
AD	ACR (L)	9.12 (1)	0.003	0.177	68.8	1.55	0.008	[0.4, 4.07]
	ACR (R)	5.71 (1)	0.017	0.120	68.9	1.50	0.023	[0.15, 3.63]
	CST (R)	5.36 (1)	0.021	0.113	60.7	2.43	0.018	[0.18, 5.47]
	PTR (R)	7.38 (1)	0.007	0.145	60.9	1.14	0.003	[0.29, 2.24]
	SFO (L)	5.09 (1)	0.024	0.109	58.3	2.11	0.019	[0.27, 4.49]

*ACR* anterior corona radiata, *AD* axial diffusivity, *CGC* ventral cingulum, *CST* corticospinal tract, *EC* external capsule, *FA* fractional anisotropy, *HC* healthy controls, *MD* mean diffusivity, *mTBI* mild traumatic brain injury, *PCR* posterior corona radiata, *PTR* posterior thalamic radiation, *RLIC* retrolenticular part of the internal capsule, *RD* radial diffusivity, *SCR* superior corona radiata, *SFO* superior fronto-occipital fasciculus, *SLF* superior longitudinal fasciculus

Regression coefficients are reported with bias-corrected and accelerated (BCa) bootstrap *p*-values and BCa 95% confidence intervals for B. Bold text indicates significance. Hemisphere: (L) left; (R) right. Units: AD, MD and RD in ×10⁻4 mm2·s⁻1; FA is unitless

### Associations between diffusion metrics and blood-based biomarkers

**Fig. 3:**
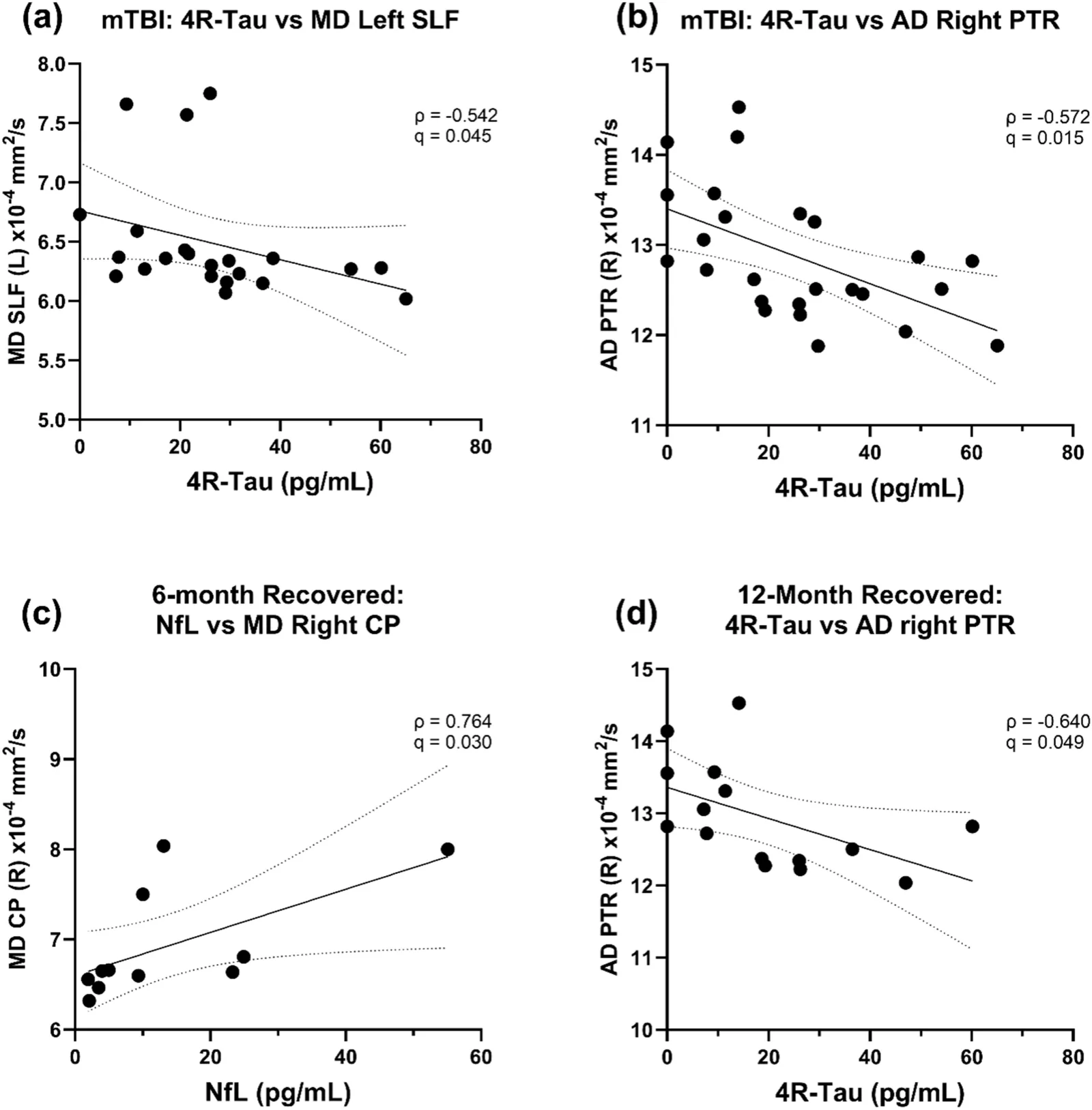
Correlations between baseline blood and diffusion MRI ROI biomarkers. Scatterplots show relationships between **(a)** 4R-tau and left SLF MD in mTBI participants, **(b)** 4R-tau and right PTR AD in mTBI participants, **(c)** NfL and right CP MD in mTBI participants who had symptomatically recovered by 6 months post-injury and, **(d)** 4R-tau and right PTR AD in mTBI participants who symptomatically recovered by 12 months post-injury. Spearman’s rank correlation coefficients (ρ) and false discovery rate-adjusted *p*-values (q) are shown. Solid lines indicated fitted linear trends and dotted lines indicate 95% confidence intervals. Abbreviations: 4R-tau, four-repeat tau; AD, axial diffusivity; CP, cerebral peduncle; MD, mean diffusivity; mTBI, mild traumatic brain injury; NfL, neurofilament light chain; PTR, posterior thalamic radiation; SLF, superior longitudinal fasciculus

Associations between blood and diffusion biomarkers were examined using Spearman’s rank-order correlations. Scatter plots of biomarker relationships are depicted in Fig. 3. After multiple comparison correction, higher baseline 4R-tau in mTBI participants was associated with lower MD within the left SLF (ρ = − 0.542, q = 0.045, Fig. 3a). This correlation was repeated with the removal of three visual outliers (7.57, 7.75, 7.66) to test the robustness of the association; following removal the association remained statistically significant though was reduced slightly in magnitude (ρ = − 0.522, q = 0.022); comparisons of the two scatterplots are visualized in Online Supplementary Resource 6. Higher 4R-tau concentration was also associated with lower AD in the right PTR (ρ = − 0.572, q = 0.015, Fig. 3b). NfL was not significantly associated with diffusion ROIs. No significant correlations between blood and diffusion biomarkers were found for control participants.

## Results: group comparisons by longitudinal recovery status

### Post-concussion symptoms at follow-up

Of the total 35 mTBI participants, 33, 31, and 31 participants had follow-up data available at 3, 6, and 12 months, respectively (2, 4, and 4 lost to follow-up at each respective timepoint). Of these participants, 39.4% (*n* = 13), 48.4% (*n* = 15), 32.3% (*n* = 10) remained symptomatic at 3, 6, and 12 months, respectively. Comparisons of PCSS scores between baseline HC and mTBI follow-up scores of recovered and symptomatic mTBI groups revealed significant group differences at 3 [H(3) = 30.67, *p* < 0.0001] 6- [H(3) = 29.45, *p* < 0.0001] and 12 months [H(3) = 28.36, *p* < 0.0001]. Individual PCSS trajectories from baseline to 12-month follow-up, plotted separately by sex are provided in Supplementary Figs. ESM5.2 and ESM5.3 to illustrate individual-level variability in recovery according to sex specific thresholds.

At all follow-up timepoints, FDR-corrected post hoc tests determined that symptomatic mTBI participants reported significantly higher symptom severity compared to both HC (3 months: q = 0.0006; 6 months: q = 0.0008; 12 months: q = 0.0004) and recovered mTBI participants (3 months: q < 0.0001; 6 months: q < 0.0001; 12 months: q < 0.0001). The total number of endorsed symptoms was also higher in symptomatic mTBI participants compared to both HC (3 months: q = 0.0017; 6 months: q = 0.0088; 12 months: q = 0.0004) and recovered mTBI participants (3 months: q < 0.0001; 6 months: q < 0.0001; 12 months: q < 0.0001).

Notably, compared to HC participants at all follow-up timepoints, recovered mTBI participants reported significantly lower symptom severity (3 months: q = 0.004; 6 months: q = 0.0042; 12 months: q = 0.0065) and symptom number (3 months: q = 0.0017; 6 months: q = 0.0088; 12 months: q = 0.0065).

### Baseline resilience and coping style by longitudinal recovery status

mTBI participants who were symptomatic mTBI at 3 months reported a significantly higher preference for passive coping at baseline compared to recovered mTBI participants (U = 66, *p* = 0.039, Online Resource 2). No significant group differences were found between follow-up groups for the BRS or other UCL subscales at any other timepoint (all *p* > 0.05)*.*

### Baseline blood-based biomarkers by longitudinal recovery status

Acute blood biomarker concentrations, stratified by longitudinal recovery status, are listed in Table 2. Significant group differences in baseline NfL were found between HC, recovered and symptomatic mTBI groups at 3, 6, and 12 months [3 months: H(2) = 12.44, *p* = 0.002; 6 months: H(2) = 6.28, *p* = 0.043; 12 months: H(2) = 8.43, *p* = 0.014].

### Acute neurofilament light chain concentration is significantly associated with symptom resolution at 3 months post-mTBI

Relative to HCs, participants who were deemed recovered at 3 months had significantly higher baseline NfL (q = 0.001). This finding was replicated at 12 months (q = 0.0078); the 6-month comparison difference was not significant with multiple comparison correction (q = 0.065). Comparing baseline NfL concentrations in the 3-month recovered vs. symptomatic mTBI participants, we found that NfL was significantly higher in the recovered mTBI group (q = 0.036). No baseline differences were observed between HC and symptomatic mTBI participants stratified by any follow-up timepoint. Due to these unexpected results, a sensitivity analysis was conducted whereby ROUT was increased from 1 to 10% to remove likely outliers and comparisons were reassessed with no changes to the pattern of results.

Given the significant differences in baseline NfL between recovered and symptomatic mTBI groups relative to subacute 3-month follow-up, univariate binary logistic regression was used to determine whether baseline NfL concentrations were associated with recovery. The overall model was significant [χ^2^(1) = 4.742, p=0.029, Nagelkerke R^2^=0.205] and correctly classified 75.9% of cases. Higher baseline NfL was associated with a reduced likelihood of being symptomatic mTBI at 3 months [Bootstrapped: B=− 1.76, OR=0.172, p=0.029, BCa 95% CI (− 4.33, − 0.54)]. Despite a group effect, baseline NfL was not significantly different between recovered and symptomatic mTBI groups at 6 and 12 months and logistic regression was, therefore, not pursued.

### Acute 4R-tau concentration is significantly associated with poor recovery at 12 months post-mTBI

Acute 4R-tau significantly differed between groups stratified by outcome status at 6 months [H(2) = 8.5, *p* = 0.014] and 12 months [H(2)=10.93, *p* = 0.004]. In post hoc comparisons, participants who were deemed symptomatic mTBI at 6 months had significantly higher baseline 4R-tau compared to HC (q=0.008), with this finding also replicated for participants meeting symptomatic mTBI criteria at 12 months compared to HC (*p* = 0.001, q=0.006), but not 3 months. Participants who were symptomatic mTBI at 12 months also had significantly higher baseline 4R-tau compared to participants who had recovered at this time (*p* = 0.008, q=0.021), however, no differences were observed between symptomatic mTBI and recovered mTBI groups relative to outcome at 3 or 6 months.

Given the differences in baseline 4R-tau between participants who recovered or remained symptomatic at 12 months post-mTBI, univariate binary logistic regression was used to determine whether acute 4R-tau was associated with long-term symptomatic status. The model was significant [χ2(1)=6.77, p=0.009, Nagelkerke R2=0.316] and correctly classified 74.1% of cases. Higher acute 4R-tau was associated with an increased likelihood of being symptomatic at 12 months [Bootstrapped B=0.068, OR=1.07, p=0.009, 95% CI (0.02, 0.28)]. Sensitivity analysis was conducted to determine the robustness of these findings (ROUT, Q=10%) with findings remaining significant.

### Baseline diffusion MRI changes by longitudinal recovery status

Group-level differences in baseline diffusion metrics are summarized in Online Resource 6. Significant effects were observed across RD, MD, FA, and AD, with post hoc comparisons indicating distinct patterns between healthy controls, recovered mTBI participants, and symptomatic mTBI participants at three-, six-, and twelve-month follow-up.

#### Radial diffusivity

Baseline RD was elevated across multiple white matter tracts (Bilateral: ACR, CGC, SFO; PCR L, TAP R, SCC) in participants who remained symptomatic at three months relative to both HC and recovered mTBI participants, with no significant group differences in RD observed beyond this timepoint.

#### Mean diffusivity

Higher baseline MD was consistently associated with better recovery outcomes. Recovered mTBI participants showed elevated MD relative to HC across association fibers at three-, six-, and 12-month follow-up (CGC R, CGH R, SLF L&R, ACR L). Recovered participants also exhibited higher baseline MD relative to mTBI participants who remained symptomatic at six and twelve months (CGC R, CGH R, CP R, SLF L).

#### Fractional anisotropy

Baseline FA was reduced in participants who remained symptomatic at three and six months post-injury compared to HC (EC R, SCR L). Acute FA was also lower in symptomatic participants relative to those who had recovered by 3 months in the right EC.

#### Axial diffusivity

Baseline AD was elevated in both recovered mTBI and symptomatic mTBI participants relative to HC across the bilateral ACR and right CST, with the pattern persisting across multiple follow-up timepoints. Recovered mTBI and symptomatic mTBI participants did not differ significantly from one another in AD for most tracts.

#### Recovery-dependent relationships between acute diffusion metrics and blood-based biomarkers

To determine recovery-specific biomarker relationships, separate correlations were conducted for recovered mTBI and symptomatic mTBI participants at each follow-up timepoint. After correction for multiple comparisons, significant relationships were only present between dMRI metrics and blood biomarkers in recovered mTBI participants. In participants who had recovered at 6-month follow-up, baseline NfL concentration was strongly positively correlated with MD in the right CP (ρ=0.764, q=0.030; Fig. 3c), meaning that NfL concentrations were higher where MD was elevated. In participants deemed recovered at 12-month follow-up, lower baseline 4R-tau was strongly negatively correlated with higher AD in the right PTR (ρ = −0.640, q=0.049; Fig. 3d), whereby lower 4R-tau concentrations were associated with higher AD. No correlations surviving correction for multiple comparisons were found between blood and diffusion biomarkers in symptomatic mTBI participants.

### Associations between acute dMRI candidates and longitudinal outcome

Acute diffusion biomarkers that showed significant differences between mTBI and HC (Table 3) were entered into bootstrapped binary logistic regressions to demonstrate case–control discrimination (Table 4). Candidate biomarkers meeting these criteria that *also* differed significantly between recovered mTBI and symptomatic mTBI groups at corresponding follow-up were entered into binary regressions to determine whether baseline metrics were associated with longitudinal symptomatic status (Online Resource 3). This two-stage procedure prioritized plausible injury-linked biomarkers and controlled model multiplicity. Biomarkers that could not discriminate between mTBI and HC groups were not analyzed further due to sample size constraints; this should not be taken as evidence against their potential prognostic value in future studies of larger cohorts.

#### Radial diffusivity

Group differences in baseline RD between recovered mTBI and symptomatic mTBI participants were observed in the left PCR, left RLIC, and right SLF relative to three-month recovery status. Despite these group-level differences, baseline RD in these tracts was not significantly associated with recovery outcome in logistic regression models.

#### Mean diffusivity

Baseline MD within the left SLF, group differences found at 6 months yielded a significant model [χ2(1)=6.66, p=0.010, Nagelkerke R2=0.320] that correctly classified 70.8% of cases. Higher baseline MD was associated with reduced likelihood of being symptomatic at 6 months post-injury [Bootstrap: B=−3.78, *p* = 0.03, BCa 95% CI(− 8.93,−1.92)]. At 12 months, the model was also significant [χ2(1)=7.41, *p* = 0.010, Nagelkerke R2=0.360] and correctly classified 66.7% of cases; higher baseline MD was associated with being symptomatic at 12 months post-mTBI [Bootstrap: B=−6.97, *p* = 0.03, BCa 95% CI(− 14.80,−3.60)]. Additional MD ROIs were not associated with outcome at any follow-up.

#### Fractional anisotropy

Group differences in baseline FA in the right EC were assessed at 3 months; however, no association with symptomatic status at follow-up was found [χ2(1)=1.24, p=0.266].

#### Axial diffusivity

Despite significant group differences over multiple tracts and timepoints, baseline AD was not associated with symptomatic mTBI status at follow-up for any ROI.

### Moderating influence of resilience and coping style

Moderation analyses examined whether resilience and coping styles influenced the relationship between acute biomarker concentrations relative to longitudinal outcome status in the mTBI group. Conditional effects are reported as ±1 SD from the mean. Given the exploratory nature of these analyses and the sample size, significant and trend-level effects are reported for hypothesis-generating, rather than confirmatory purposes.

#### Passive coping style is a significant moderator of the relationship between acute NfL concentration and 3-month recovery status

The passive reaction coping style significantly moderated the relationship between acute NfL and outcome at 3 months [− 2LL=22.99, LR χ2(3)=13.5, p=0.004, Nagelkerke R^2^=0.53]; the interaction between passive reaction coping and NfL was also significant [b=− 9.7, SE:4.92, Wald p=0.048; LR χ2(1)=8.72, p=0.003]. Conditional effects analysis indicated that higher NfL was associated with lower odds of 3-month symptomatic status that approached significance at the mean (b=− 6.46, p=0.052) and high passive reaction coping use (b=− 12.64, p=0.047), but not at low levels (b=− 0.29, p=0.8). Moderation models of NfL and 3-month outcome were not significant for the BRS or the other UCL subscales (reassuring thought coping, emotional expression, social support seeking, avoidance, active tackling, palliative coping).

#### Reassuring thought coping style is a significant moderator of the relationship between acute 4R-tau concentration and poor outcome at 12 months post-mTBI

The reassuring thought coping style significantly moderated the relationship between acute 4R-tau and 12-month symptomatic status; the overall model was significant [− 2LL=13.82; LR χ2(3)=18.28, *p* < 0.001, Nagelkerke R2=0.721]. The interaction term between 4R-tau and reassuring thought coping was also significant [b=− 0.32, SE=0.162, Wald *p* = 0.049, LR χ2(1)=9.69, *p *= 0.002]. Conditional effects analysis indicated that higher baseline 4R-tau was associated with greater odds of being symptomatic at 12 months when acute reports of reassuring thought coping were low [− 1 SD: (b=0.27, *p* = 0.04, 95% CI (0.01, 0.52)]; effects were not significant at mean (x̄: b=0.09, *p* = 0.11) or high use (+ 1 SD: b=− 0.10, *p* = 0.20). Moderation models of 4R-tau and 12-month outcome were not significant for the BRS or other UCL subscales.

#### Relationships between mean diffusivity and longitudinal outcome were not moderated by resilience or coping style

At 6 months post-injury, neither coping style or resilience moderated the relationship between MD in the SLF and outcome. However, omnibus models were significant for passive reaction coping [− 2LL=19.67, LR χ2(3)=12.17, *p* = 0.01], emotional expression coping [− 2LL=21.41, LR χ2(3)=10.43, *p* = 0.02] and social support seeking [− 2LL=23.91, LR χ2(3)=7.93, *p* = 0.048], suggesting individual effects of these factors rather than an interaction between them. At 12 months post-injury, overall model fit was significant for emotional expression coping [− 2LL=19.52, LR χ2(3)=10.20, *p* = 0.017], palliative coping [− 2LL=19.34, LR χ2(3)=10.38, *p* = 0.016], passive reaction coping [− 2LL=9.98, LR χ2(3)=19.74, *p* < 0.001], reassuring thoughts [− 2LL=18.97, LR χ2(3)=10.75, *p* = 0.013] and BRS resilience [− 2LL=20.83, BRS: LR χ2(3)=8.89, *p* = 0.031]. Coping style and BRS scores did not demonstrate significant interactions with left SLF MD in any model (all, *p* > 0.05).

## Discussion

Integration of multimodal biomarkers alongside clinical characteristics and psychosocial assessments to better characterize mTBI is increasingly recommended in current international research and clinical guidelines [2, 40]. Consistent with this, our study provides initial exploratory, hypothesis-generating findings in a modest yet demographically diverse and heterogeneous sample. We examined whether acute blood and diffusion MRI biomarkers of WM injury distinguished mTBI from healthy controls, differentiated longitudinal recovery trajectories, and were moderated by resilience and coping style. Acute mTBI was characterized by elevations in NfL and 4R-tau alongside widespread diffusivity changes within projection and association fibers, with distinct acute biomarker profiles across recovery trajectories. Coping style, rather than resilience, moderated psychological processes and biomarkers of WM injury in relation to longitudinal outcome. Together, our findings suggest biological phenotypes of recovery spanning molecular, microstructural, and psychological domains that may inform prognosis and targeted follow-up.

Uncomplicated mTBI is more likely to result in subtle disruption of the axonal cytoskeleton rather than the frank axotomy observed in more severe injuries though these axons are still highly vulnerable to secondary processes that perpetuate ionic disturbances and impair transport [76]. NfL is considered a sensitive proxy for axonal integrity due to its high abundance within large-caliber axons [77, 78] and near-exclusive expression within the CNS [79]. Injury-induced dysregulation of intra- and extracellular calcium homeostasis activates calpains and other proteases, contributing to mitochondrial and synaptic dysfunction and propagating secondary mechanisms [76, 80, 81]. NfL is thought to enter the peripheral circulation via calpain-mediated proteolysis of neurofilament sidearms, promoting filament disassembly and release of NfL into the extracellular fluid where it is subsequently cleared into the blood [82, 83]. Baseline NfL concentrations in our mTBI group exceeded those of controls, consistent with previous findings [84–87], though the absence of concurrent BD-tau elevations suggests subtle axonal perturbation rather than overt degeneration. Several studies have now linked acute NfL elevation to prolonged symptoms [84, 85, 88–90]. We did not identify associations between NfL and persisting symptoms; rather, *higher* NfL concentrations were associated with 3-month recovery. Symptom burden was substantially lower in recovered mTBI participants relative to both control and symptomatic mTBI participants at this time, mirroring symptom divergence already present at baseline. On average, blood samples were collected 5.6 days post-injury, a timepoint consistent with the ascending phase of NfL elevation reported in the literature [85, 91, 92]. Peak concentrations of NfL have been observed in concussed athletes at approximately six days post-injury [85, 92] suggesting that our samples were collected at a timepoint of robust signal rather than post-peak decline. The absence of serial sampling nonetheless limits characterization of individual NfL trajectories in this sample.

Our observation that higher NfL concentrations were associated with favorable outcomes presents an apparent paradox. Here, lower symptom burden and NfL elevation in recovered mTBI participants may not reflect overt pathological damage, but rather transient cytoskeleton disruption [76, 93, 94]. Neurofilament proteins are known to undergo proactive structural remodeling in response to physiological stress [93, 94], which may provide an alternative explanation for the elevated concentrations observed in participants reporting symptomatic recovery. This is in line with our observed differences in baseline MD between symptomatic and recovered mTBI participants, with those who went on to recover having pronounced increases in MD across several tracts. This may reflect expansion of the extracellular space alongside subtle microstructural intracellular changes, without overt demyelination or degeneration [95]. Palacios et al. reported similar relationships in which higher acute MD was associated with favorable outcomes at 6 months post-injury [96]. In the present study, participants who recovered at 6 months demonstrated positive correlations between acute MD in the right CP and NfL concentration. No such relationship between biomarkers was evident in the cohort of participants who remained symptomatic at this time. These convergent NfL and MD findings suggest that a more pronounced neurobiological response may facilitate, rather than impede downstream recovery, and add nuance to findings that have predominantly linked biomarker elevation to poorer outcome [84, 89, 97, 98].

This adaptive interpretation stands in contrast to the acute biomarker profile of those who remained symptomatic, for whom 4R-tau elevation was an acute blood biomarker feature. Attempts to restore homeostasis may involve upregulation of cytoskeletal proteins that confer stability and facilitate axonal transport [82, 94, 99]. Ratios of 3R- to 4R-tau isoforms are roughly equal in the adult CNS; disturbance in this ratio is associated with several neurodegenerative diseases [99–101]. The additional microtubule-binding repeat domain (R) in 4R-tau isoforms increases its affinity for tubulin, effectively preventing the disassembly of microtubules [99, 102] while simultaneously increasing its propensity for hyper-phosphorylation [103–105]. Upon detachment from microtubules, 4R-tau becomes vulnerable to depolymerization and capable of initiating a pathological intracellular cascade [99, 101, 105, 106]. Free cytoplasmic 4R-tau exerts a strong inhibitory effect on axonal mitochondrial transport [107], destabilizes endoplasmic reticulum–mitochondrial calcium regulation [108], impairs astrocytic glutamate clearance and inhibits neuronal firing [109]— each of which may compromise post-injury recovery. Acute elevations in 4R-tau in peripheral blood found in this study imply its release from the intracellular compartment, with possible initiation of cytoskeletal perturbation as a result. These pathobiological consequences may explain why 4R-tau, but not NfL or BD-tau, were associated with symptomatic mTBI status at 12 months. Unlike NfL, whose elevation may reflect a transient injury process, and BD-tau which may indicate more overt structural injury, 4R-tau may catalyze a specific mechanism of ongoing dysfunction that persists longitudinally and actively impedes recovery. GFAP and UCH-L1, both of which did not show significant elevation at the five-day sampling timepoint, a finding consistent with the rapid post-injury kinetics of these markers that both peak within 24–48 h and rapidly decline thereafter [87, 110, 111], suggesting that concentrations had likely normalized prior to sampling rather than reflecting a null biological response.

BD-tau is exclusive to the CNS [112, 113] and has shown promise as a marker of axonal injury in more severe TBI [113, 114]. At this time, neither BD-tau nor 4R-tau has been evaluated as candidate blood biomarkers in human mTBI. Matrix- and substrate-specific differences limit direct comparisons of BD- and 4R-tau concentrations and, the present study cannot directly inform the ratio of 3R:4R. However, selective 4R-tau elevation in the absence of BD-tau suggests that isoform specific biology rather than overall abundance alone, may be relevant to prolonged recovery This raises the possibility that disruption to tau-isoform balance may provide prognostic information in milder injuries, where overt structural degeneration is less apparent. Future studies using assays capable of directly quantifying the 3R:4R tau ratio are required to determine whether acute tau-isoform imbalance predicts persistent symptoms, reflects a transient injury response, or indicates broader vulnerability to tau-associated neurodegenerative processes. Our findings specific to those who experienced prolonged recovery raises the question of whether acute isoform imbalance may persist and progress over longer timepoints. Studies of chronic traumatic encephalopathy (CTE) have documented disruption of the 3R:4R-tau ratio, where 4R isoforms predominate in early disease stages and later shift to 3R dominance [115]. Though the link between mTBI, PPCS and CTE is yet to be fully elucidated, convergence of shared mechanisms supports the notion of a neurodegenerative continuum associated with tau-isoform imbalance [106]. Acute concentrations were only associated with outcome at the 12-month follow-up, by which time the sample size had reduced. Attrition and potential response bias, including self-selection by more symptomatic mTBI patients limits the generalizability of these estimates [16, 116]. Determining whether 4R-tau concentrations continue to rise, plateau or normalize in the months following injury would strengthen its candidacy as a marker of prolonged symptom burden. Given the exploratory nature of this work, future studies in larger prospective cohorts and parallel investigation beyond canonical axonal biomarkers may inform the causative mechanism of 4R-tau elevation we observed.

Divergent changes in acute WM diffusivity in participants with persisting symptoms were characterized by widespread increases in RD, particularly within projection and association fibers. Radial diffusivity describes changes orthogonal to fiber orientation in which elevations are hypothetically associated with myelin sheath alteration [22]. Given the acute imaging timepoint and exclusion of complicated mTBI, overt demyelination would be unlikely; the sensitivity of RD to extracellular fluid imbalance [22] suggests ionic disturbance and neuroinflammatory processes are more probable [22]. This interpretation is consistent with the acute pathophysiological profile of uncomplicated mTBI, wherein subtle injury processes predominate over overt structural degradation [117]. Affected tracts, such as the corona radiata and cingulum, are long-range fibers that are particularly susceptible to injury with these tracts consistently implicated regions in dMRI studies of mTBI [29, 118, 119]. While all mTBI participants demonstrated altered acute diffusivity in these regions, alterations were more pronounced in those who experienced prolonged symptoms at 3 months, but not 6 and 12 months. Whether participants were classified into recovered mTBI or symptomatic mTBI groups varied according to their responses at each follow-up; group membership was, therefore, not static, limiting our inference over time.

Our observation that those with persisting symptoms exhibited more pronounced acute RD elevations suggests that the degree of initial extracellular disruption in these biomechanically vulnerable tracts may differentiate recovery trajectories, at least in the short term. Higher acute RD in symptomatic mTBI participants is consistent with more extensive localized ionic disequilibrium and extracellular fluid accumulation in the early post-injury phase, processes that may reflect a greater burden of axonal injury [22, 35, 117]. An absence of RD group differences at 6- and 12-month follow-ups suggests that this metric may represent an acute-phase process that either resolves or becomes less discriminative over time. This temporal pattern is also reflected in our logistic regression analyses, where RD was not significantly associated with symptomatic mTBI status despite group-level separation, highlighting the distinction between-group-level statistical differences and individual-level prognostic utility. Secondary injury processes are temporally dynamic, and our single acute timepoint may have impacted our ability to relate blood biomarker and diffusivity changes longitudinally. This may explain the lack of significant correlations between biomarkers in participants who remained symptomatic mTBI at specific follow-up timepoints. Though temporally distinct, co-occurrence of elevated RD and elevated peripheral 4R-tau in participants with persisting symptoms likely reflects converging signatures of the same injury process rather than a causal relationship between these measures; RD captures microscopic tissue disruption, while peripheral 4R-tau reflects molecular-level cytoskeletal compromise. That both were elevated in participants who did not recover symptomatically suggests a greater burden of initial injury. This is further supported by reports of higher baseline PCSS severity scores in this subgroup and is in line with previous findings [17, 120].

Within the broader mTBI group, higher 4R-tau was associated with lower MD in the left SLF and lower AD in the right posterior thalamic radiation; a pattern of restricted diffusion which may reflect acute neuroinflammation [121]. Although symptoms cannot be mapped directly to these individual ROIs, their involvement of major projection and association pathways suggests that these findings may reflect broader disruption to white matter systems relevant to sensorimotor and cognitive integration. Although most correlations did not reach significance, the direction of association between 4R-tau and MD across ROIs was consistently negative in mTBI participants and reversed for controls, suggestive of injury-specific relationships. The novelty of 4R-tau as a biomarker precludes firm inference to the biological basis or clinical significance associated with this interaction. The absence of significant relationships between diffusion metrics and NfL in the broader mTBI group was unexpected, but has been reported previously and may relate to lower injury severity [122, 123]. When correlations were examined by recovery status, significant biomarker relationships were only present for recovered mTBI participants; no significant blood–diffusion correlations were identified in symptomatic mTBI participants. This was contrary to our hypothesis that persisting symptoms would be accompanied by stronger cross-modal associations, reflecting a coherent multimodal injury signature. Our findings suggest instead that coherent cross-modal relationships may characterize an organized, proportional injury response in those who recover. In symptomatic mTBI participants, the absence of coherent correlations may reflect greater pathophysiological heterogeneity, the influence of additional non-white matter processes, or a decoupling of biomarker trajectories that contributes to, or results from, impaired recovery. Multimodal biomarker studies in aging cohorts have demonstrated that blood-based and neuroimaging measures, even when sensitive to overlapping pathology, load onto distinct latent constructs that covary only under specific biological conditions or are influenced by additional psychosocial factors [124] – a pattern that may partly explain the absent cross-modal correlations in symptomatic mTBI participants here.

Blood biomarkers, rather than diffusion metrics, appear to be more susceptible to influences of reassuring thought and passive reaction coping styles. Passive reaction coping strategies, such as withdrawal or cognitive distancing, are considered maladaptive [53] and have previously been associated with chronic post-injury fatigue [125]. In the present study, higher use of passive coping style was associated with both elevated plasma NfL and recovery, a pattern that runs counter to the established maladaptive framing. Though our sample size limits robust inference, one possibility is that passive coping strategies may reduce cognitive demand on an acutely injured brain, acting as enforced rest rather than maladaptive avoidance. Alternatively, passive coping may reflect a stable premorbid disposition rather than an injury-driven response. Spikman and colleagues found that passive coping style was consistent across the first 6 months post-injury and was associated with lower chronic cortisol levels both pre- and post-mTBI [126], suggesting it captures an enduring individual characteristic. Our associations between passive reaction coping, elevated NfL and recovery may reflect pre-existing differences in stress biology that co-occur with a more robust acute biological response that merit replication in larger, similarly demographically diverse cohorts. Reassuring thought coping altered the association of acute 4R-tau and recovery at 12 months post-injury: lower preference increased the influence of 4R-tau, increasing the likelihood of experiencing prolonged symptoms. This moderating effect may partially reflect the greater baseline symptom burden observed in symptomatic mTBI participants, which could influence both coping appraisal and symptom reporting independent of biomarker levels [127, 128]. Whether elevated 4R-tau in this context is causative or consequential prolonged recovery remains to be determined. Conceptually, these findings may point to a scenario in which less adaptive coping profiles amplify the biological impact of injury. Rather than acute burden, it may be that chronicity of symptoms and reliance on less effective coping profiles perpetuate unfavorable biological conditions, given that the brain engages similar pathways in response to psychological and physiological threat [129, 130]. Reduced use of reassuring thoughts and greater reliance on passive reaction strategies may sustain maladaptive biological responses, thereby exacerbating existing unfavorable biological profiles and the likelihood of prolonged recovery [129, 131, 132]. With respect to diffusion biomarkers, although several tests reached significance, we did not find moderating effects, indicating that coping style and/or resilience independently contribute to outcome rather than influencing WM microstructure. This distinction implies that blood biomarkers may be more amenable to the influence of psychosocial buffering compared to diffusion metrics, or that diffusion metrics are less responsive to these influences in the acute post-injury window.

### Limitations and future directions

We acknowledge the statistical power constraints of sample size in the current study and emphasize that our results should be considered exploratory and theory building. Beyond sample size, a fundamental caveat of the current work is the inherent subjectivity of outcome classification. A truly objective biological signature of mTBI cannot be disentangled from the bias imposed by self-report as inter-individual perceptions of symptomatic burden inevitably limit the objectivity of outcome classification, even when considered alongside physiological data. Multimodal biomarker integration and its inclusion alongside resilience and coping were employed to reduce this subjectivity, though self-reported symptoms remained an indispensable factor. In cases of mTBI, where no pathognomonic biomarker exists, acute and longitudinal symptomology remains a key accessible indicator of individual experience and functional output, particularly in contexts wherein advanced diagnostic infrastructure is unavailable. Likewise, our sample was diverse in terms of age, sex, injury mechanism and inclusive of those with pre-existing physiological and psychological health conditions. These factors may better reflect the clinical heterogeneity of real-world mTBI populations, but may have also introduced additional variability that obscured more specific injury-related effects. Use of a single, acute data point also restricted our ability to determine whether observed biological changes evolve or become self-limiting over time, and how this relates to symptom burden; this likewise applies to resilience and coping style, which may also be dynamic across the recovery trajectory. The current cohort lacked pre-injury assessment of coping and resilience, constraining the extent to which moderation effects reflect stable pre-existing traits rather than post-injury adaptation. Prospective recruitment in high-risk cohorts where mTBI is plausible, but unpredictable, athlete populations for example, would allow for more objective inference of pre-injury baselines. Such designs would, however, reduce the clinical generalizability of findings to the broader mTBI population in which falls and motor vehicle incidents dominate. Given the dynamic nature of the secondary injury cascade, longitudinal follow-up in a larger cohort that included blood biomarker and diffusion collection would substantially strengthen the inferences posited here and better characterize the temporal evolution of secondary processes and their relationship to long-term outcomes.

## Conclusion

Our findings support the use of a multimodal approach to mTBI diagnosis and prognosis. Outcome trajectory was defined by divergent acute WM responses: NfL and MD elevation in those who recovered at follow-up and 4R-tau and RD increase in those who experienced persisting symptoms with diffusion abnormalities most evident projection fibers, particularly the anterior corona radiata. Acute phenotypes were exacerbated or attenuated through the influence of passive reaction and reassuring-thought-based coping, suggesting a convergence of biological and psychological mechanisms in shaping longitudinal outcomes. While exploratory in nature, our findings highlight both novel biomarker avenues and potentially modifiable psychological constructs that warrant investigation in larger, prospective cohorts.

## Supplementary Information

Below is the link to the electronic supplementary material.

Supplementary file1 (PDF 91 KB)

Supplementary file2 (PDF 188 KB)

Supplementary file3 (PDF 105 KB)

Supplementary file4 (PDF 386 KB)

Supplementary file5 (PDF 557 KB)

Supplementary file6 (PDF 177 KB)

## Data Availability

The datasets generated and/or analyzed during the current study are available from the corresponding author on reasonable request.
