# Supplementary material for: Divergent recovery trajectories after mild traumatic brain injury are characterized by distinct acute profiles of neurofilament light, 4R-tau, and white matter diffusivity
Source: J Neurol. 2026 Aug 25;273(9):548. doi: 10.1007/s00415-026-14060-0 (PMC13506629; doi:10.1007/s00415-026-14060-0)
Supplement: Supplementary file 1 [file 415_2026_14060_MOESM1_ESM.pdf]

Supplementary Table ESM1: Abbreviations of the 48 white matter tracts from the JHU ICBM-DTI-81 atlas.

**Abbreviation**	**White Matter Tract**
ACR	Anterior Corona Radiata
ALIC	Anterior Limb of Internal Capsule
BCC	Body of Corpus Callosum
CGC	Cingulum (Ventral)
CGH	Cingulum (Hippocampal)
CP	Cerebral Peduncle
CST	Corticospinal Tract
EC	External Capsule
Fx	Fornix (Column and Body of Fornix)
Fx-ST	Fornix/Stria Terminals
GCC	Genu of Corpus Callosum
ICP	Inferior Cerebellar Peduncle
MCP	Middle Cerebellar Peduncle
ML	Medial Lemniscus
PCR	Posterior Corona Radiata
PCT	Pontine Crossing Tract
PLIC	Posterior Limb of the Internal Capsule
PTR	Posterior Thalamic Radiation
RLIC	Retrolenticular Part of the Internal Capsule
SCC	Splenium of Corpus Callosum
SCP	Superior Cerebellar Peduncle
SCR	Superior Corona Radiata
SFO	Superior Fronto-Occipital Fasciculus
SLF	Superior Longitudinal Fasciculus
SS	Sagittal Stratum
TAP	Tapetum
UNC	Uncinate Fasciculus

Abbreviations correspond to white matter tracts defined by the JHU ICBM-DTI-81 white matter atlas [69, 70].

[1]Oishi K, Zilles K, Amunts K, et al. Human brain white matter atlas: Identification and assignment of common anatomical structures in superficial white matter. NeuroImage. 2008;43(3):447-457. doi:10.1016/j.neuroimage.2008.07.009

[2]Mori S, Oishi K, Jiang H, et al. Stereotaxic white matter atlas based on diffusion tensor imaging in an ICBM template. NeuroImage. 2008;40(2):570-582. doi:10.1016/j.neuroimage.2007.12.035
