# Supplementary material for: Divergent recovery trajectories after mild traumatic brain injury are characterized by distinct acute profiles of neurofilament light, 4R-tau, and white matter diffusivity
Source: J Neurol. 2026 Aug 25;273(9):548. doi: 10.1007/s00415-026-14060-0 (PMC13506629; doi:10.1007/s00415-026-14060-0)
Supplement: Supplementary file 2 [file 415_2026_14060_MOESM2_ESM.pdf]

Supplementary Table ESM2: Participant demographics, injury characteristics, and post-concussion symptom burden at 3-, 6-, and 12-month follow-ups, stratified by recovery status.

	3-Months		6-Months		12-Months	
Mean ± SD or n (%)	*Recovered (n=20)*	*Symptomatic (n=13)*	*Recovered (n=16)*	*Symptomatic (n=15)*	*Recovered (n=21)*	*Symptomatic (n=10)*
Demographics						
Female sex	5 (25)	7 (53.8)	6 (37.5)	7 (46.7)	7 (33.3)	5 (50)
Age in years	34.8 ± 11.8	41.7 ± 16.5	34.1 ± 11.1	42.9 ± 16.4	34.8 ± 12.6	43.7 ± 16.7
BMI	24.4 ± 2.6	25.3 ± 3.4	24.8 ± 1.8	24.5 ± 4	24.7 ± 2.4	25.1 ± 4.0
Years of Education	14.4 ± 2.4	14.1 ± 1.9	14.3 ± 2.2	13.6 ± 2.4	13.9 ± 2.2	14.4 ± 2.0
Regular Exercise	19 (95)	12 (92.3)	15 (93.8)	13 (86.7)	21 (100)	9 (90)
Injury Characteristics						
Sports-related	**15 (75)**	**4 (30.8)**	**11 (68.8)**	**5 (33.3)**	**16 (76.2)**	**2 (20)**
Falls	2 (10)	5 (38.5)	**1 (5.3)**	**7 (53.8)**	**1 (4.8)**	**6 (60)**
MVA	3 (15)	3 (23.1)	4 (25)	2 (13.3)	4 (19)	1 (10)
Struck object	0 (0)	1 (7.7)	0 (0)	1 (6.7)	0 (0)	1 (10)
Lost Consciousness						
Yes	8 (40)	3 (23.1)	5 (31.3)	6 (40)	8 (38.1)	2 (20)
Unsure	5 (25)	3 (23.1)	4 (25)	2 (13.3)	5 (23.8)	2 (20)
Posttraumatic amnesia						
Yes	11 (55)	7 (53.8)	6 (37.5)	11 (73.3)	11 (52.4)	6 (60)
Unsure	1 (5)	0 (0)	1 (6.3)	0 (0)	1 (4.8)	0 (0)
Non-concussive injuries	8 (40)	7 (53.8)	7 (43.8)	8 (53.3)	10 (47.6)	4 (40)
Pre-existing conditions						
Prior mTBI	14 (70)	8 (61.5)	9 (56.3)	11 (73.3)	12 (57.1)	8 (80)
Migraine	4 (20)	2 (15.4)	2 (12.5)	3 (20)	4 (19)	2 (20)
Dyslexia	2 (10)	0 (0)	2 (10.5)	0 (0)	2 (9.5)	0 (0)
ADHD	0 (0)	2 (15.4)	1 (6.3)	1 (6.7)	1 (4.8)	1 (10)
Insomnia	0 (0)	1 (7.7)	0 (0)	1 (6.7)	0 (0)	1 (10)
Depression	2 (10)	3 (23.1)	1 (6.3)	5 (3.3)	3 (14.3)	2 (20)
Anxiety	0 (0)	3 (23.1)	1 (6.3)	2 (13.3)	1 (4.8)	2 (20)
PTSD	0 (0)	1 (7.7)	0 (0)	1 (6.7)	0 (0)	1 (10)
PCSS						
Severity	**1.5 ± 1.6**	**20.3 ± 12.6**	**1.4 ± 2.0**	**18.1 ± 11.1**	**1.7 ± 2.1**	**28.1 ± 17**
Number	**0.9 ± 1.1**	**9.1 ± 3.8**	**1.1 ± 1.6**	**7.6 ± 3.7**	**1.2 ± 1.5**	**12.8 ± 3.4**
Brief Resilience Scale	3.9 ± 0.5	3.7 ± 0.6	3.8 ± 0.6	3.8 ± 0.6	3.9 ± 0.5	3.7 ± 0.7
Utrecht Coping List						
Active Tackling	2.7 ± 0.5	2.7 ± 0.4	2.7 ± 0.5	2.7 ± 0.3	2.7 ± 0.4	2.8 ± 0.4
Palliative Response	2.2 ± 0.4	2.4 ± 0.5	2.3 ± 0.5	2.3 ± 0.5	2.2 ± 0.5	2.5 ± 0.5
Avoidance/Waiting	2.0 ± 0.3	2.2 ± 0.4	2.0 ± 0.3	2.2 ± 0.4	2.0 ± 0.4	2.2 ± 0.5
Social Support	2.4 ± 0.5	2.4 ± 0.7	2.4 ± 0.4	2.4 ± 0.7	2.3 ± 0.5	2.6 ± 0.7

Passive Response	**1.4 ± 0.4**	**1.8 ± 0.6**	1.5 ± 0.4	1.8 ± 0.5	1.5 ± 0.4	1.9 ± 0.6
Emotional Expression	2.1 ± 0.6	2.1 ± 0.4	2.0 ± 0.5	2.1 ± 0.4	2.1 ± 0.5	2.3 ± 0.3

Supplementary Table S2: Participant demographics, injury characteristics, and post-concussion symptom burden at 3-, 6-, and 12-month follow-ups, stratified by recovery status. Values are presented as mean ± standard deviation or number (percentage). Bolded values indicate statistically significant differences between recovered and PPCS groups at each timepoint ($p<0.05$). **Abbreviations:** ADHD, attention-deficit hyperactivity disorder; BMI, body mass index; mTBI, mild traumatic brain injury; MVA, motor vehicle accident; PCSS, post-concussion symptom scale; PPCS, persisting-post concussion symptoms; PTSD, post-traumatic stress disorder.
