# Supplementary material for: Divergent recovery trajectories after mild traumatic brain injury are characterized by distinct acute profiles of neurofilament light, 4R-tau, and white matter diffusivity
Source: J Neurol. 2026 Aug 25;273(9):548. doi: 10.1007/s00415-026-14060-0 (PMC13506629; doi:10.1007/s00415-026-14060-0)
Supplement: Supplementary file 3 [file 415_2026_14060_MOESM3_ESM.pdf]

Supplementary Table ESM3: Candidate baseline diffusion biomarkers associated with symptomatic status at follow-up

Metric	Tract	Follow-up	χ^2(df)	P (Omnibus)	R^2	Correct (%)	B (BCa)	P (BCa)	Bca 95% CI [lower, upper]
RD	PCR (L)	3	2.15 (1)	0.14	0.100	65.5	0.92	0.24	[-0.58, 4.49]
	RLIC (L)	3	1.92 (1)	0.17	0.090	58.6	1.27	0.18	[-1.29, 5.57]
	SLF (R)	3	1.92 (1)	0.17	0.090	62.1	1.42	0.13	[-1.95, 5.47]
MD	ACR (L)	6	2.65 (1)	0.10	0.140	66.7	-1.42	0.06	[-3.37, -0.14]
	CGC (R)	12	3.88 (1)	0.05	0.203	62.5	-4.61	0.26	[-19.6, -2.02]
	CGC (R)	3	2.62 (1)	0.11	0.130	65.4	-2.83	0.12	[-12.29, -0.72]
	CGC (R)	6	7.64 (1)	0.01	0.364	70.8	-8.12	0.16	[-28.04, -3.69]
	SLF (L)	6	6.66 (1)	0.01	0.324	70.8	-3.78	0.03	[-8.97, -1.93]
	SLF (L)	12	7.41 (1)	0.01	0.362	66.7	-6.97	0.04	[-15.59, -3.31]
FA	EC (R)	3	1.24 (1)	0.26	0.054	63.6	-22.1	0.28	[-67.6, 15.8]
AD	ACR (L)	3	0.15 (1)	0.70	0.007	55.2	0.22	0.71	[-1.2, 1.48]
	ACR (L)	6	1.08 (1)	0.30	0.052	63.0	0.62	0.34	[-1.04, 5.6]
	ACR (L)	12	0.20 (1)	0.65	0.010	63.0	0.27	0.66	[-1.39, 2.97]
	ACR (R)	3	0.15 (1)	0.70	0.008	53.8	-0.33	0.71	[-2.64, 1.11]
	ACR (R)	6	1.55 (1)	0.21	0.083	62.5	1.17	0.21	[-1.32, 4.54]
	ACR (R)	12	1.22 (1)	0.27	0.068	62.5	1.05	0.26	[-1.45, 4.62]
	CST (R)	3	0.82 (1)	0.37	0.042	61.5	-1.37	0.36	[-6.28, 2.69]
	CST (R)	6	0.28 (1)	0.60	0.015	45.8	-0.85	0.61	[-5.53, 3.67]
	CST (R)	12	0.002 (1)	0.97	0.000	62.5	0.07	0.97	[-4.28, 6.82]
	SFO (L)	6	0.32 (1)	0.57	0.018	54.2	-0.79	0.59	[-5.41, 2.91]
	SFO (L)	12	0.13 (1)	0.72	0.007	62.5	-0.52	0.71	[-3.78, 2.58]

Baseline diffusion measures used in univariate binary logistic regression to assess whether acute biomarkers were associated with symptoms at follow-up. For each tract and metric, model performance is summarised by χ^2(df), omnibus p, Nagelkerke R^2, and classification accuracy. Regression coefficients are reported with bootstrap inference (BCa p values and BCa 95% confidence intervals for B). Hemisphere: (L) left; (R) right. Units: AD, MD and RD in $\times 10^{-4}$ $mm^2 \cdot s^{-1}$; FA is unitless. Bold text indicates $p<0.05$ (BCa bootstrap p values).

Abbreviations: ACR, anterior corona radiata; AD, axial diffusivity; CST, corticospinal tract; EC, external capsule; FA, fractional anisotropy; MD, mean diffusivity; PCR, posterior corona radiata; RD, radial diffusivity; RLIC, retrolenticular part of the internal capsule; SFO, superior fronto occipital fasciculus; SLF, superior longitudinal fasciculus.
