# Supplementary material for: Divergent recovery trajectories after mild traumatic brain injury are characterized by distinct acute profiles of neurofilament light, 4R-tau, and white matter diffusivity
Source: J Neurol. 2026 Aug 25;273(9):548. doi: 10.1007/s00415-026-14060-0 (PMC13506629; doi:10.1007/s00415-026-14060-0)
Supplement: Supplementary file 4 [file 415_2026_14060_MOESM4_ESM.pdf]

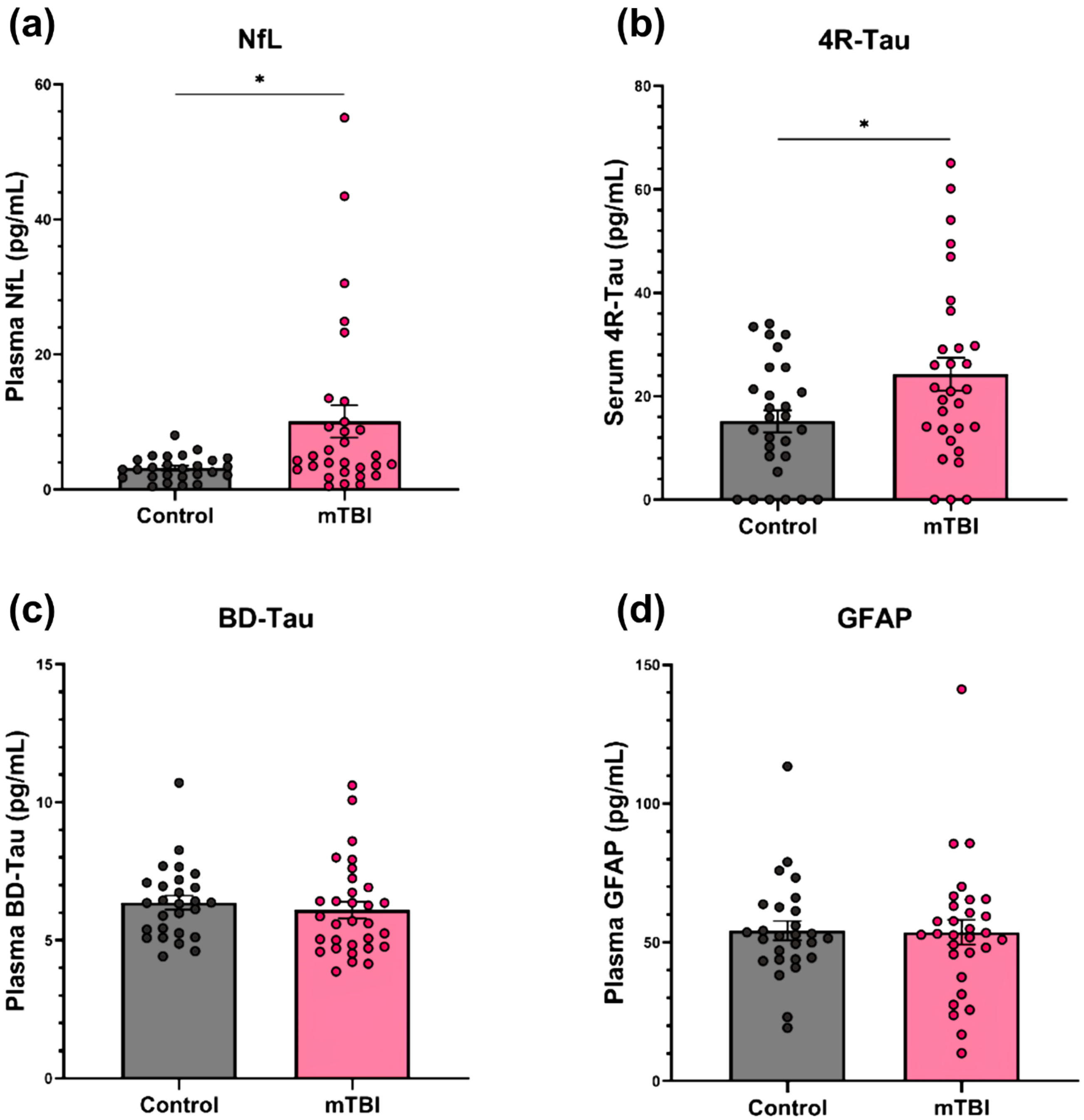

Supplementary Fig ESM4: Baseline concentrations of **(a)** NfL, **(b)** 4R-tau, **(c)** BD-tau and **(d)** GFAP for healthy controls and acute mild traumatic brain injury participants.

Group comparisons were performed using the Mann Whitney U test, with significance set at $p<0.05$. An asterisk indicates a statistically significant difference. Points represent individual participants, and bars represent mean ± SEM measured in pg/mL. NfL and 4R-tau concentrations were significantly elevated in the mTBI group relative to control; BD-tau and GFAP did not differ between groups. 4R-tau, four-repeat tau; BD-tau, brain-derived tau; GFAP, glial fibrillary acidic protein; mTBI, mild traumatic brain injury; NfL, neurofilament light chain.
