# Supplementary material for: Divergent recovery trajectories after mild traumatic brain injury are characterized by distinct acute profiles of neurofilament light, 4R-tau, and white matter diffusivity
Source: J Neurol. 2026 Aug 25;273(9):548. doi: 10.1007/s00415-026-14060-0 (PMC13506629; doi:10.1007/s00415-026-14060-0)
Supplement: Supplementary file 5 [file 415_2026_14060_MOESM5_ESM.pdf]

Online Supplementary Resource 5:

Additional analyses and results

Results: Comparison of Baseline mTBI vs Healthy Controls
Demographic and Clinical Characteristics

The most common mechanism of injury was sports-related concussion (SRC; 51.1%, n=20) followed by fall (22.9%, n=8). mTBI and HC groups were well matched in terms of age, sex and pre-existing medical conditions but differed with respect to years of education (mTBI: 14.0 ± 2.3; HC: 15.5 ± 1.8 years; p=0.03), self-reported concussion history (p<0.001) and BMI (mTBI: 24.8 ± 2.8; HC: 23.1 ± 3.2; p=0.02). Those who experienced a SRC were significantly younger than those from other mechanisms (SRC: 32.5 ± 12.2; non-SRC: 44.4 ± 13.0 years; p=0.002). PTA(<24 hours) and LOC (<30 minutes) were confirmed by 54.3% (n=19) and 31.4% (n=11) participants, respectively.

Acute Post-Concussion Symptoms

At baseline, fatigue was the most frequently reported symptom, experienced by 89% (n=31) of mTBI participants; 59% (n=20) of HC participants also reported fatigue, though at a lower average severity compared to mTBI participants. Concentration difficulties, reported by 74% of mTBI participants had the highest mean severity score of all symptoms (1.97 ± 1.8).

Diffusion MRI Differences Between HC and mTBI Groups

Radial Diffusivity

Compared to HC, mTBI participants had significantly elevated RD within the left posterior corona radiata (PCR: U=340, p=0.023) and right anterior corona radiata (ACR: U=331.5, p=0.015). RD was also significantly elevated following mTBI within the left retrolenticular limb of the internal capsule (RLIC: U=325.5, p=0.011) and bilaterally in the superior longitudinal fasciculus (right SLF: U=315, p=0.007; left SLF: U=315.5, p=0.008).

Mean Diffusivity

Acute MD elevations were found in the bilateral ACR (left: U=310, p=0.03; right: U=286.5, p=0.022), left SLF (U=308, p=0.029) and right ventral cingulum (CGC: U=273.5, p=0.006) compared to HC.

Fractional Anisotropy

Acute FA was significantly lower in the mTBI group compared to HC in the left SCR [t(62.82)=2.06, p=0.044], left PCR [t(61)= 2.13, p=0.037] and left EC [t(61)=2.21, p=0.03].

Axial Diffusivity

Compared to HC, the mTBI group exhibited higher AD within the bilateral ACR (left: U=298, p=0.004; right: U=287, p=0.002), right corticospinal tract (CST: U=322.5, p=0.011), right posterior thalamic radiation (PTR: t(54.3)=2.70, p=0.009) and left superior fronto-occipital fasciculus (SFO: U=309.5, p=0.017).

Results: Group Comparisons by Longitudinal Recovery Status
Demographic and Clinical Characteristics

Within mTBI follow-up groups (recovered mTBI , symptomatic mTBI), non-SRC injuries were associated with poorer recovery at all follow-up timepoints (3mo: χ^2=6.31, p=0.01; 6mo: χ^2=4.39, p=0.04; 12mo: χ^2=8.78, p=0.003). No significant differences were observed between recovered mTBI and symptomatic mTBI groups with respect to demographics, pre-existing conditions, or other injury characteristics. Additional demographic information, acute UCL and BRS findings are listed in Table

1; demographic information, follow-up PCSS scores, BRS and UCL scores split into follow-up recovery group are listed in Online Resource 2.

Post-Concussion Symptoms at Follow-Up

At 3-month follow-up, the most reported symptom among recovered mTBI participants was headache (n=3; 15%), while 77% (n=10) of symptomatic mTBI participants reported fatigue, memory problems, and concentration difficulties alike. At 6-months, fatigue was the most endorsed symptom among both recovered mTBI (n=4; 25%) and symptomatic mTBI (n=10; 66.7%) participants. At 12-months, 19% (n=4), of recovered mTBI participants reported fatigue while 100% of symptomatic mTBI participants reported memory problems (n=10) and 80% reported fatigue (n=8).

Associations Between Diffusion Metrics and Blood-based Biomarkers

higher baseline 4R-tau in mTBI participants was associated with lower MD within the left SLF (ρ=-0.542, q=0.045).We repeated the Spearman correlation after excluding the three potentially influential observations (7.57, 7.75, 7.66). The close association remained statistically significant but was reduced in magnitude (ρ=-0.522, 95% CI [-0.795, -0.075], q=0.022) and have attached the scatter plots for each with, and without the three values. A comparison between graphs with and without outliers is depicted below:

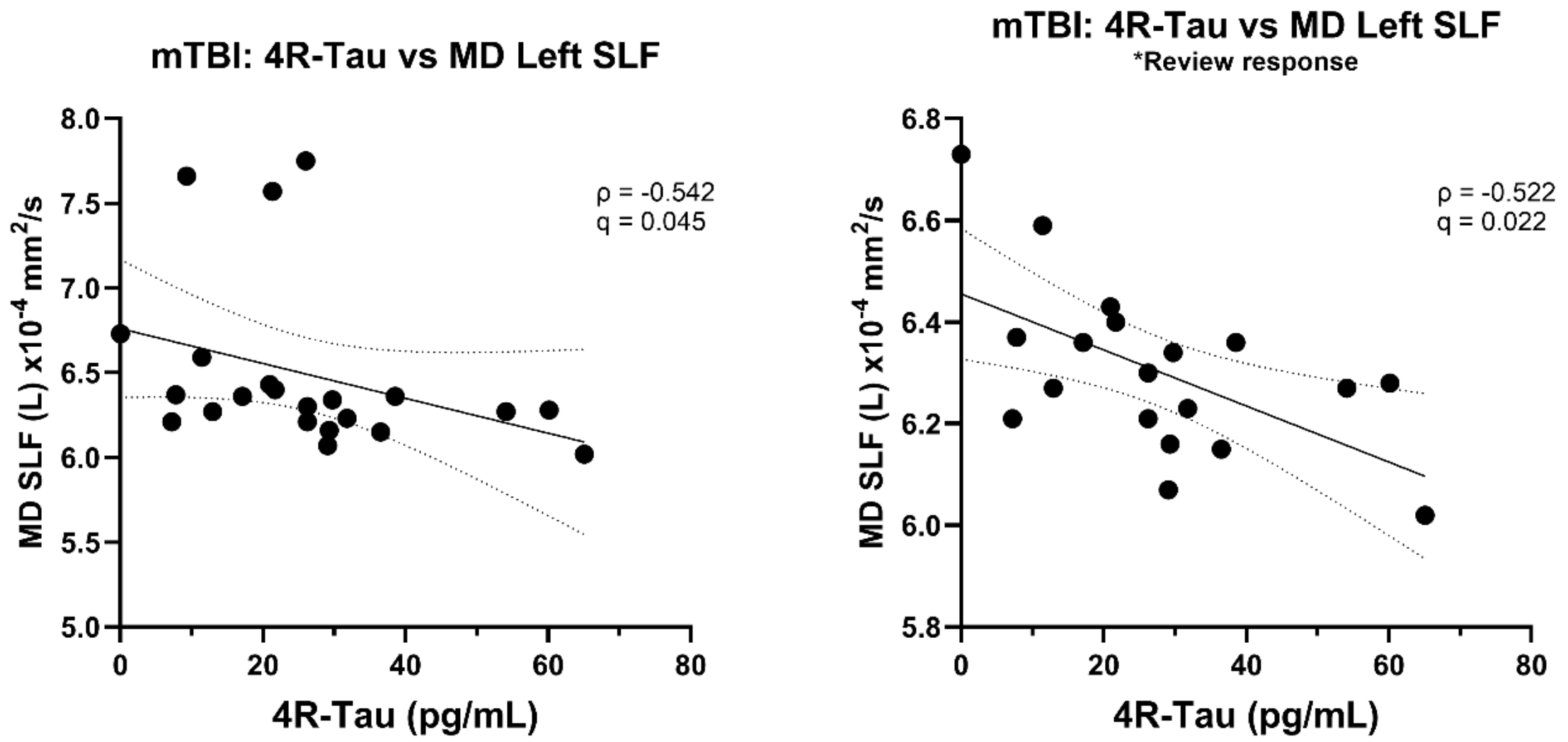

Fig. ESM5.1: A comparison of the correlation between baseline 4R-tau and MD in the left SLF, before and after exclusion of the three potentially influential observations.

Individual PCSS Trajectories by Sex

To further examine the pattern of symptomatic recovery over time, particularly the increase in symptomatic participants observed at 6-month follow-up, individual PCSS trajectories were plotted for each participant from baseline to 12-month follow-up, separated by sex. These plots illustrate both the variability in individual recovery paths and the differing data availability across timepoints, as not all participants contributed PCSS data at every follow-up. Trajectories are shown relative to the sex-specific recovery threshold (PCSS <6 for males, <7 for females).

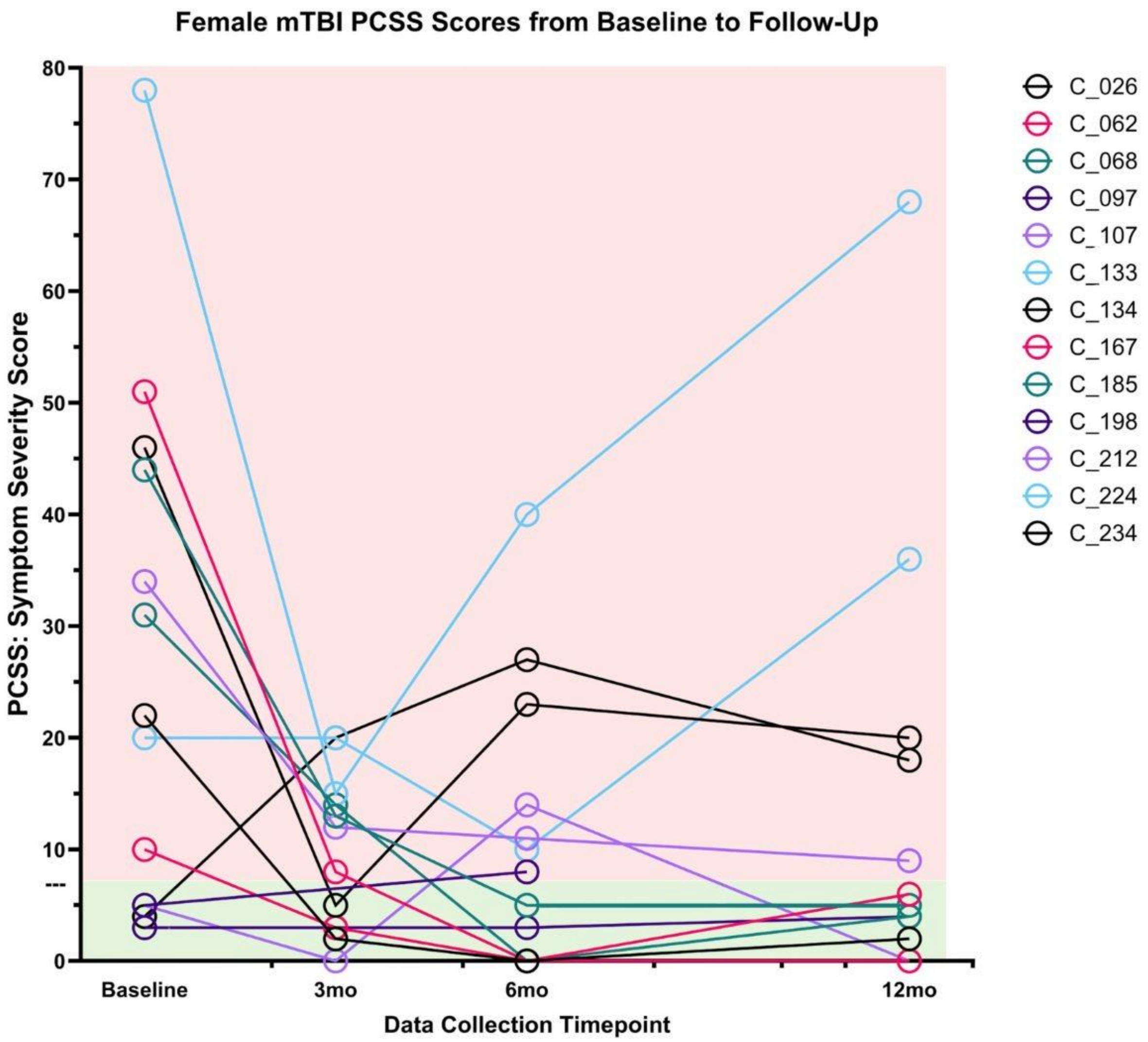

Fig. ESM5.2 Individual PCSS trajectories for female mTBI participants from baseline to 12-month follow-up. Each line represents a single participant, connecting available PCSS scores across timepoints; gaps indicate missing follow-up data. The dashed line denotes the sex-specific recovery threshold (PCSS <7 for females); shaded regions indicate symptomatic (red, PCSS ≥7) and recovered (green, PCSS <7) ranges. PCSS, Post-Concussion Symptom Scale.

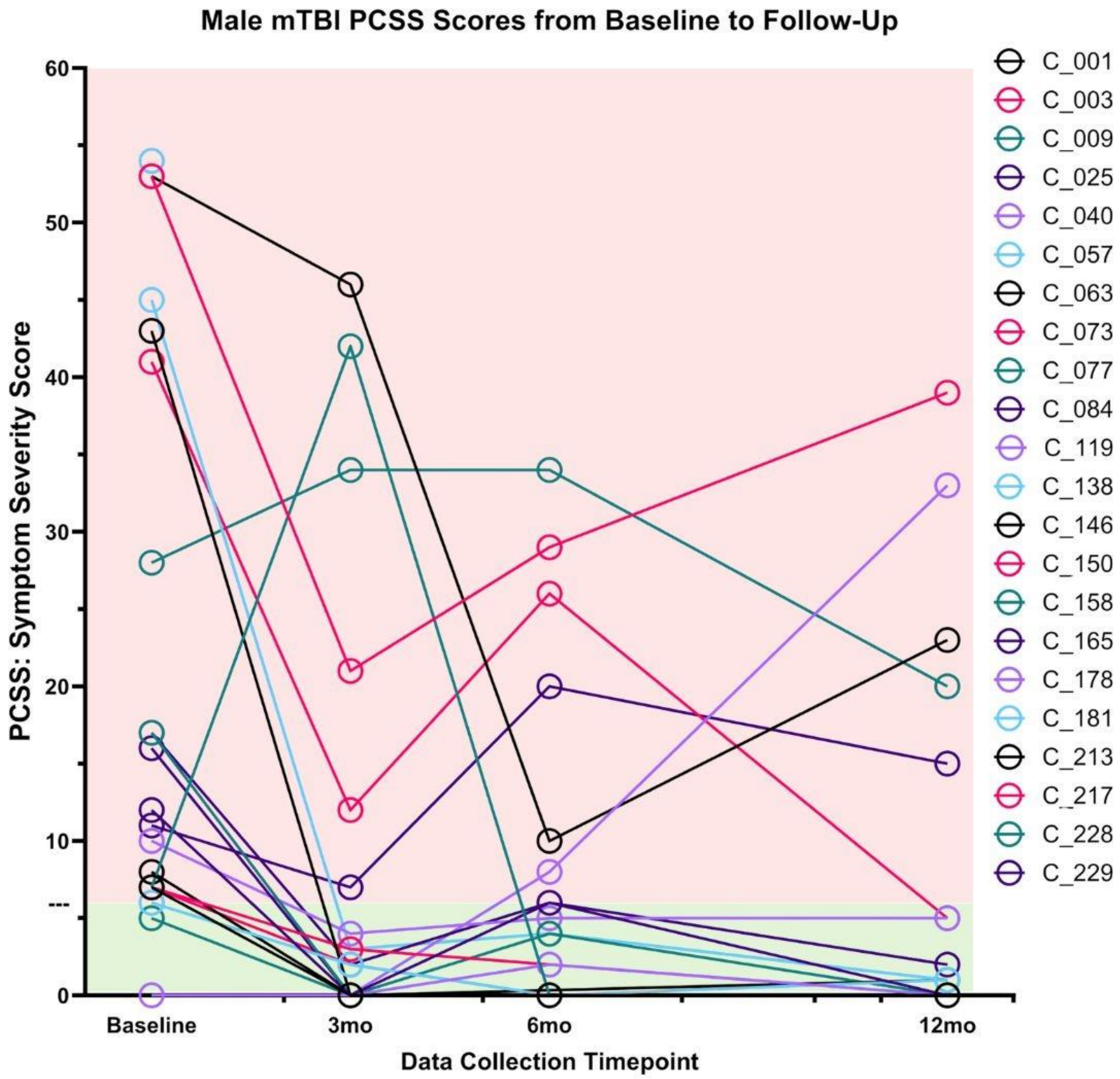

Fig. ESM5.3 Individual PCSS trajectories for male mTBI participants from baseline to 12-month follow-up. Each line represents a single participant, connecting available PCSS scores across timepoints; gaps indicate missing follow-up data. The dashed line denotes the sex-specific recovery threshold (PCSS <6 for males); shaded regions indicate symptomatic (red, PCSS ≥6) and recovered (green, PCSS <6) ranges. PCSS, Post-Concussion Symptom Scale.

To assess the robustness of baseline NfL group differences and associated correlations with diffusion metrics, sensitivity analyses were conducted with visual outliers removed. Baseline plasma NfL was compared across symptom groups (Control, Recovered, Symptomatic) at both 3-month and 6-month follow-up, with and without outlier removal. Correlations between baseline NfL and mean diffusivity (MD) in the right cerebral peduncle were also examined among participants recovered at 6 months, with and without outlier removal."

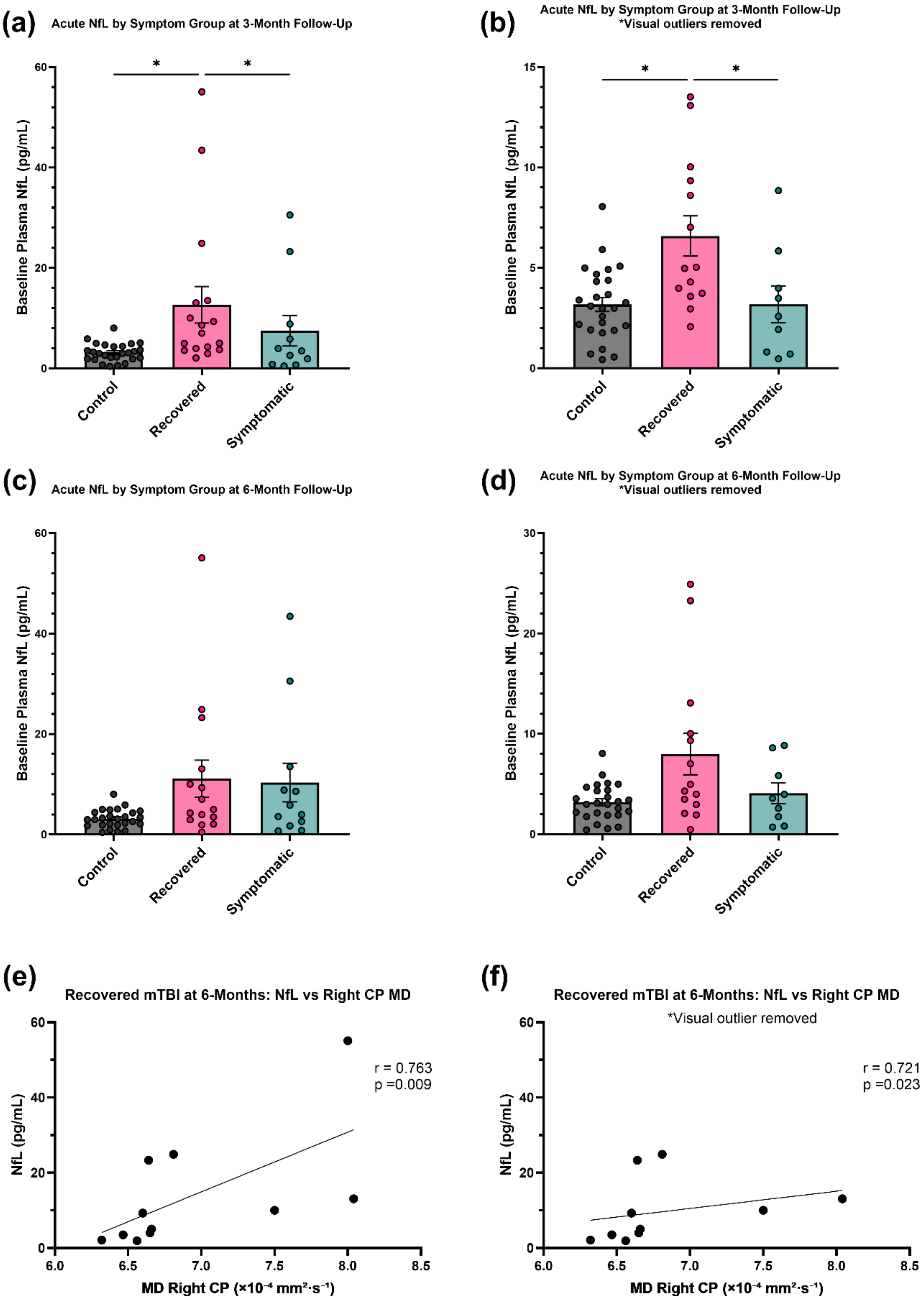

Fig. ESM5.4 Sensitivity of baseline NfL group differences and NfL-diffusion correlations to outlier removal. (a) Baseline plasma NfL by 3-month symptom group (Control, Recovered, Symptomatic). (b) Baseline plasma NfL by 3-month symptom group with visual outliers removed. (c) Baseline plasma NfL by 6-month symptom group. (d) Baseline plasma NfL by 6-month symptom group with visual outliers removed. (e) Correlation between baseline NfL and MD in the right cerebral peduncle (CP) in participants recovered at 6-months. (f) Correlation between baseline NfL and MD in the right CP in participants recovered at 6-months, with visual outlier removed. Group differences and correlation coefficients (r) with associated p-values are shown; *$p<0.05$. CP, cerebral peduncle; MD, mean diffusivity; NfL, neurofilament light chain.
