# Supplementary material for: Divergent recovery trajectories after mild traumatic brain injury are characterized by distinct acute profiles of neurofilament light, 4R-tau, and white matter diffusivity
Source: J Neurol. 2026 Aug 25;273(9):548. doi: 10.1007/s00415-026-14060-0 (PMC13506629; doi:10.1007/s00415-026-14060-0)
Supplement: Supplementary file 6 [file 415_2026_14060_MOESM6_ESM.pdf]

Online Supplementary Resource 6: Group differences in diffusion metrics across white matter tracts at three-, six-, and twelve-month follow-up.

Metric	*Tract*	*Follow-Up (Months)*	*Group Effect (p)*	**HC vs REC** *Difference*	*q*	**HC vs SYM** *Difference*	*q*	**REC vs SYM** *Difference*	*q*
**RD**	*ACR L*	3	0.011*	-	0.23	HC < SYM	0.008*	REC < SYM	0.014*
	ACR R	3	0.005*	-	0.155	HC < SYM	0.001*	REC < SYM	0.015*
	PCR L	3	0.014*	-	0.269	HC < SYM	0.008*	-	0.077
	CGC L	3	0.01*	-	0.569	HC < SYM	0.022*	REC < SYM	0.042*
	CGC R	3	0.032*	-	0.345	HC < SYM	0.004*	REC < SYM	0.006*
	SFO L	3	0.026*	-	0.156	HC < SYM	0.013*	REC < SYM	0.002*
	SFO R	3	0.025*	-	0.195	HC < SYM	0.01*	REC < SYM	0.01*
	TAP R	3	0.006*	-	0.272	HC < SYM	0.002*	REC < SYM	0.002*
	SCC	3	0.011*	-	0.239	HC < SYM	0.004*	REC < SYM	0.004*
**MD**	*CGC R*	3	0.001*	HC < REC	0.006	-	0.199	-	0.198
		6	0.005*	HC < REC	0.003	-	0.312	SYM < REC	0.033*
		12	0.012*	HC < REC	0.006	-	0.293	-	0.146
	CGH R	6	0.020*	-	0.082	-	0.071	SYM < REC	0.013*
	CP R	6	0.022*	HC < REC	0.01	-	0.163	SYM < REC	0.009*
	SLF L	6	0.025*	HC < REC	0.014	-	0.285	-	0.108
		12	0.042*	HC < REC	0.007	-	0.612	SYM < REC	0.044*
	SLF R	12	0.020*	HC < REC	0.012	-	0.554	-	0.076
	ACR L	6	0.043*	-	0.129	-	0.280	-	0.229
**FA**	*EC R*	3	0.039*	HC > REC	0.038	SYM < HC	0.015*	SYM < REC	0.015*
		6	0.013*	-	0.762	SYM < HC	0.012*	-	0.088
		12	0.045*	-	0.395	-	0.059	-	0.880
	SCR L	3	0.042*	-	0.881	SYM < HC	0.038*	-	0.484
		6	0.049*	-	0.699	SYM < HC	0.044*	-	0.485
**AD**	*ACR L*	3	0.021*	HC < REC	0.016*	HC < SYM	0.016*	-	0.266
		6	0.001*	-	0.161	HC < SYM	0.010*	-	0.161
		12	0.039*	-	0.107	-	0.107	-	0.845
	ACR R	3	0.015*	HC < REC	0.014*	HC < SYM	0.014*	-	0.335
		6	0.012*	-	0.147	HC < SYM	0.011*	-	0.147
		12	0.024*	-	0.086	-	0.052	-	0.086
	CST R	3	0.028*	HC < REC	0.031*	-	0.083	-	0.459
		12	0.025*	-	0.094	-	0.094	-	0.929
	PTR R	6	0.040*	-	0.178	-	0.058	-	0.553
		12	0.048*	-	0.051	-	0.393	-	0.492
	SFO L	6	0.022*	-	0.122	-	0.122	-	0.846
		12	0.016*	-	0.081	-	0.081	-	0.830

Group comparisons were conducted using Kruskal-Wallis or one-way ANOVA, as appropriate to data distribution. Multiple comparisons were controlled using the two-stage linear step-up procedure of Benjamini, Krieger, and Yekutieli. Pairwise comparisons are presented for healthy controls (HC) versus recovered mTBI (REC), healthy controls (HC) versus symptomatic mTBI (SYM), and recovered mTBI (REC) versus symptomatic mTBI (SYM). Bold p- and q-values indicate statistical significance (p<0.05 or q<0.05). Directional comparisons indicate the group with the lower diffusion value. A dash indicates that the pairwise comparison did not survive correction for multiple comparisons.
Abbreviations: ACR L/R, anterior corona radiata left/right; AD, axial diffusivity; CGC L/R, cingulate gyrus cingulum left/right; CGH R, hippocampal cingulum right; CP R, cerebral peduncle right; CST R, corticospinal tract right; EC R, external capsule right; FA, fractional anisotropy; HC, healthy controls; MD, mean diffusivity; PCR L, posterior corona radiata left; PTR R, posterior thalamic radiation right; RD, radial diffusivity; REC, recovered mTBI ; SCC, splenium of corpus callosum; SCR L, superior corona radiata left; SFO L/R, superior fronto-occipital fasciculus left/right; SLF L/R, superior longitudinal fasciculus left/right; SYM, symptomatic mTBI; TAP R, tapetum right; q, two-stage linear step-up corrected p-value.
